# Untargeted Metabolomic and Lipidomic Profiles of Gingival Crevicular Fluid in the Context of Periodontitis

**DOI:** 10.1111/jcpe.70105

**Published:** 2026-02-09

**Authors:** Caroline H. Henderzahs, Chiaki Yamada, Alexandr Morozov, Thelmalane Yalartai, Vanchit John, Hawra AlQallaf, Alexandru Movila

**Affiliations:** ^1^ Department of Periodontology Indiana University School of Dentistry Indianapolis Indiana USA; ^2^ Department of Oral Biology and Comprehensive Science Indiana University School of Dentistry Indianapolis Indiana USA; ^3^ Indiana Center for Musculoskeletal Health Indiana University School of Medicine Indianapolis Indiana USA; ^4^ Richard L. Roudebush VA Medical Center Indianapolis Indiana USA; ^5^ Institute of Zoology, State University of Moldova Chisinau Republic of Moldova; ^6^ Department of Biology St. Catherine University St. Paul Minnesota USA

**Keywords:** clinical study, gingival crevicular fluid, periodontitis, semisupervised deep learning, untargeted metabolomics

## Abstract

**Aim(s):**

This cross‐sectional clinical study aimed to explore the untargeted metabolomic and lipidomic profiles in gingival crevicular fluid (GCF) obtained from healthy individuals and patients with periodontitis.

**Materials and Methods:**

GCF was collected from 17 periodontally healthy and 19 periodontitis patients. For the unbiased characterisation of positively and negatively charged polar metabolite and lipid compounds, we employed hydrophilic interaction and reversed‐phase liquid chromatography/mass spectrometry platforms, followed by a semisupervised deep learning‐based approach for metabolomic peak curation and data analysis.

**Results:**

A total of 256 metabolites were identified with Metabolomics Standards Initiative (MSI) confidence Levels 1–3, including 198 elevated and 58 diminished compounds in periodontal lesions (*q* < 0.05, |log_2_‐transformed Fold Change (FC)| > 1). Periodontitis samples exhibited a significant positive accumulation of purine degradation and ceramide metabolites, along with a negative regulation of oxy fatty acids metabolism. Additionally, we observed a significant increase in established periodontitis biomarkers, including N‐acetylneuraminic acid, citrulline and 2‐pyrrolidineacetic acid. The study also characterised distinct differences in bacterial and fungal metabolite profiles between the healthy and diseased samples.

**Conclusions:**

These findings suggest that untargeted metabolomic screening of GCF may significantly improve our understanding of biochemical changes between healthy tissue and periodontitis. This knowledge is pivotal for the development of a precision paradigm in periodontitis.

## 1 Introduction

Periodontitis is a chronic inflammatory disease that may lead to tooth loss and serve as a risk factor for various systemic diseases, for example, diabetes mellitus, cardiovascular disease and dementia (Albandar et al. 2018).

Several clinical measurements, including probing depth (PD), clinical attachment level (CAL), bleeding on probing (BOP) and the severity of radiographic bone loss (RBL), provide essential information about the status of the tissues surrounding the tooth. However, they are subject to technical biases, which may hinder the accurate evaluation of periodontitis severity (Dursun and Tözüm 2016). Therefore, there is an immediate need to improve current periodontitis diagnostic protocols.

Emerging clinical studies suggest that screening for polar metabolites and lipids in gingival crevicular fluid (GCF) holds considerable promise for the diagnosis of periodontitis, complementing established clinical examination procedures (Baima et al. 2021; Barnes et al. 2009). Additionally, pathologic changes observed in GCF are more accurately correlated with the signature metabolites of periodontitis (Condor et al. 2025).

Liquid chromatography/mass spectrometry (LC/MS) platforms are widely used for characterisation of metabolomic signatures via targeted and/or untargeted assays (Allwood et al. 2021). Targeted metabolomics focuses on the analysis of specific categories of metabolites with greater selectivity and sensitivity (Roberts et al. 2012). Untargeted metabolomics is a hypothesis‐generating approach and attempts to analyse all detectable metabolites from the sample, including unknown compounds (Di Minno et al. 2021). Notably, untargeted metabolomics often employs machine learning approaches for large‐scale data analysis (Sirocchi et al. 2024).

Due to the scarcity of untargeted metabolomic datasets, our understanding of the crosstalk between clinical metabolomic signatures of GCF and periodontitis remains limited (Baima et al. 2021; Brito et al. 2022). Therefore, this clinical study aimed to enhance our understanding of the metabolomic and lipidomic untargeted signatures in GCF from periodontitis and healthy periodontium samples using a combination of LC/MS platforms, followed by semisupervised deep learning approaches.

## 2 Materials and Methods

This study was approved by the Institutional Review Board of Indiana University at Indianapolis (IRB #19010). Two GCF samples free of extrasulcular contaminants were obtained from the same intrasulcular site using absorbent points (Dentsply Sirona). Pooled samples were immediately frozen in phosphate‐buffered solution and stored at −80°C until further processing.

The mobile phases for polar metabolites and lipids were analysed by Hydrophilic Interaction LC/MS and Reversed‐Phase LC/MS assays, respectively. Metabolites were structurally identified using Metabolomics Standards Initiative (MSI) Levels (1–4) and standardised across assays. Unbiased LC/MS metabolomic and lipidomic detection of metabolite signals was performed using a semisupervised deep learning‐based PeakDetective approach implemented in Python3.10.

The Log_2_‐transformed fold change (FC) with median‐normalised metabolite intensities was used for null hypothesis testing. To capture metabolomic group separation between healthy and periodontitis samples, principal component analysis (PCA) was performed using the scikit‐learn machine learning package available in Python3.10. Statistical significance was defined as *p* < 0.05. The resulting *p*‐values were corrected for multiple testing using the Benjamini–Hochberg false discovery rate (FDR; *q*) procedure, as implemented in Python's statsmodels. Significance thresholds were set to *q* < 0.05 and |Log_2_(FC)| > 1.0.

Clinical inclusion and exclusion criteria for sample collection, as well as a detailed untargeted metabolomic protocol, are provided in the Supporting Information and Materials and Methods section.

## 3 Results

### 3.1 Study Population

In this study, samples designed as healthy were represented by 70.59% of individuals with clinically healthy gums and 29.41% with gingivitis. Periodontitis samples were primarily represented by individuals diagnosed as Stage II Grade A (5.26%), Stage III Grade A (10.53%), Stage III Grade B (26.32%), Stage III Grade C (10.53%), Stage IV Grade B (21.05%) and Stage IV Grade C (26.32%). Periodontal parameters, including the total number of teeth, PDs, CALs and BOP, gender and smoking status, are represented in Tables 1 and S1. Notably, the periodontitis samples were more frequently associated with posterior teeth than with anterior teeth (Table 2).

**TABLE 1:** Demographics, periodontal diagnosis and clinical periodontal parameters of the 36 cases included in the untargeted metabolomic analysis of gingival crevicular fluid by LC/MS platforms.^a^

	Healthy controls	Periodontitis
Demographic variables		
Number of patients (%)	17 (47.22)	19 (52.78)
Age (years)	50.39 ± 23.38	58.63 ± 15.06
Gender (%)	M: 7 (19.44) F: 10 (27.78)	M: 9 (25.0) F: 10 (27.78)
Smoking status (%)	0.00	47.37
Periodontal diagnosis		
Clinical gingival health (%)	12 (70.59)	0.00
Dental biofilm induced gingivitis (%)	5 (29.41)	0.00
Stage I Grade A, B, C (%)	0.00	0.00
Stage II Grade A (%)	0.00	1 (5.26)
Stage II Grade B, C (%)	0.00	0.00
Stage III Grade A (%)	0.00	2 (10.53)
Stage III Grade B (%)	0.00	5 (26.32)
Stage III Grade C (%)	0.00	2 (10.53)
Stage IV Grade A (%)	0.00	0.00
Stage IV Grade B (%)	0.00	4 (21.05)
Stage IV Grade C (%)	0.00	5 (26.32)
Periodontal parameters		
Whole mouth		
Total number of teeth/participant	27.39 ± 2.93	22.84 ± 6.36
Total PDs ≥ 5 mm/participant	0 ± 0	23.68 ± 21.93
PD (mm)	2.00 ± 0.38	3.17 ± 0.76
CAL (mm)	2.12 ± 0.38	3.52 ± 0.77
BOP (%)	8.83 ± 8.57	36.94 ± 26.42
Sample site		
PD (mm)	1.65 ± 0.61	7.47 ± 1.71
CAL (mm)	1.82 ± 0.73	7.79 ± 1.81
BOP (%)	0.00	100.00

*Note*: All data (excluding number of patients, gender, smoking status and periodontal diagnosis) are given as mean ± SD.

Abbreviations: BOP, bleeding on probing; CAL, clinical attachment level; F, female; M, male; PD, periodontal probing depth.

^a^ One sample from the healthy controls group was excluded from the study.

**TABLE 2:** Gingival crevicular fluid samples collected from maxillary and mandibular anterior and posterior teeth.

Sample site	Maxillary anterior (%)	Maxillary posterior (%)	Mandibular anterior (%)	Mandibular posterior (%)	Total sample
Healthy controls	1 (5.9)	3 (17.6)	0 (0.0)	13 (76.5)	17
Periodontitis	4 (21.1)	8 (42.1)	0 (0.0)	7 (36.8)	19

Altogether, 36 samples from 17 healthy (7 males and 10 females) and 19 periodontitis (9 males and 10 females) were submitted for untargeted metabolomic screening (Table S1).

### 3.2 Metabolomic Divergences Between GCF Samples of Healthy and Periodontitis Individuals

**FIGURE 1:**
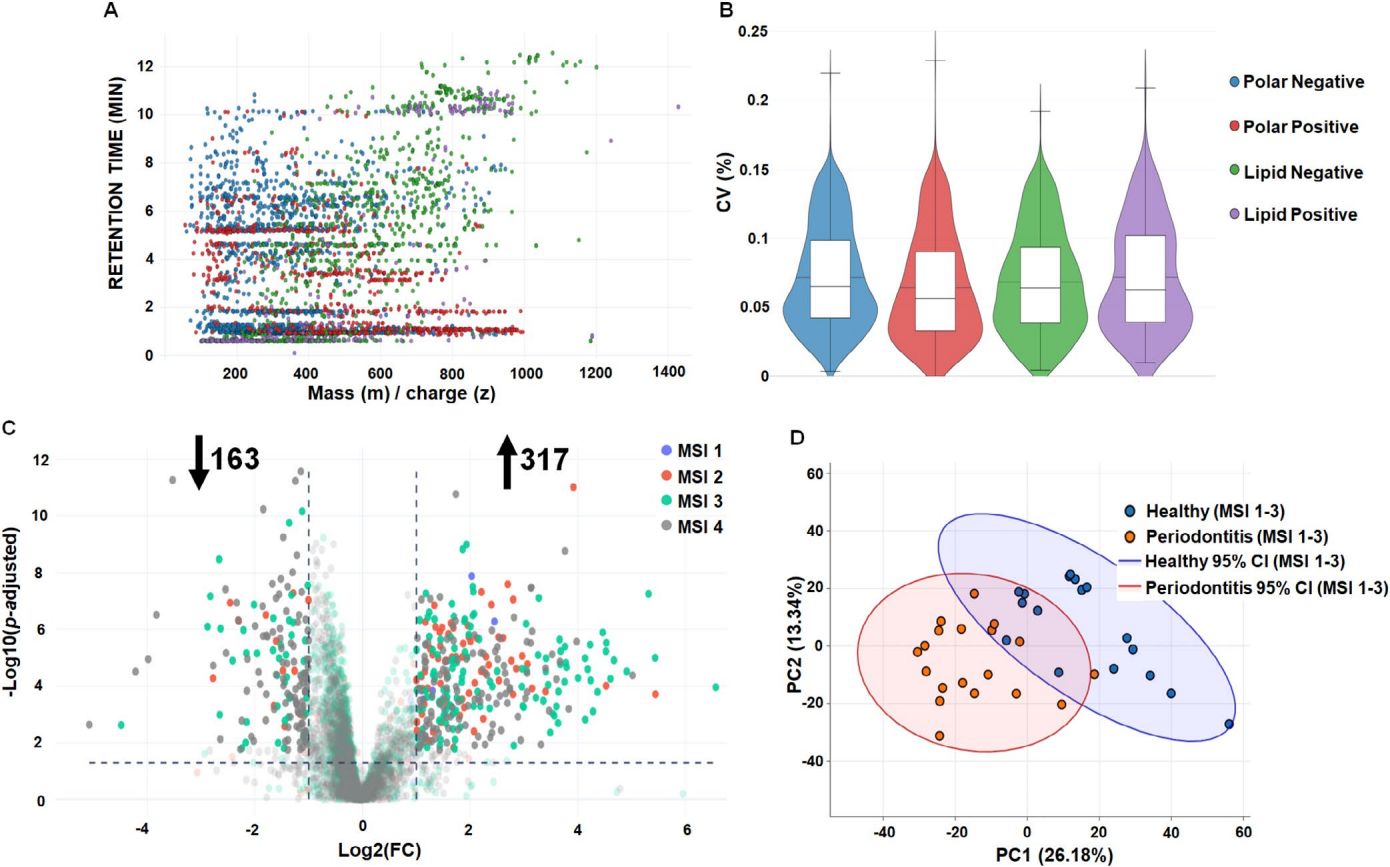
Metabolome profile summary identified in the gingival crevicular fluid (GCF) of patients with periodontitis and healthy controls using LC/MS. (A) Metabolite signals detected in GCF samples by Hydrophilic Interaction LC/MS and Reversed‐Phase LC/MS. Each metabolite signal is represented by a point on the plot representing the mass‐to‐charge ratio (m/z) and retention time coordinates of the feature. (B) Coefficients of variation (CV) across profiled metabolites. The colour of the dots corresponds to the type of assay. (C) Volcano plot of metabolites profile distributed according to the MSI with confidence Level 1–4. A total of 480 metabolites showed significant differences (*q* < 0.05 and |Log_2_ (FC)| > 1). See interactive Figure S1. (D) Differentially abundant MSI Level 1–3 metabolites in healthy (blue colour) and periodontitis (red colour) samples based on the PCA with 95% confidence interval (CI). See Table S2 and Material and Methods in Supporting Information.

We identified 4168 polar metabolite and lipid compounds in the collected GCF samples (Figure 1A). Notably, the coefficient of variation was less than 10%, indicating precise detection and data extraction (Figure 1B). Based on |Log_2_(FC)| > 1 and *q* < 0.05 selection criteria, we confirmed 317 upregulated and 163 downregulated metabolites distribution at MSI confidence Levels as Level 1–0.41%, Level 2–5.21%, Level 3–36.3% and Level 4–58.09% (Figures 1C and S1; Tables S2 and S3). Using scikit‐learn PCA algorithm, we further detected that the first principal component (PC1) accounted for 26.18% of the total variation, and the second principal component (PC2) accounted for 13.30% (Figure 1D). These data indicate that the combined use of the GCF collection technique, untargeted LC/MS platforms and robust data analysis successfully and comprehensively characterised the metabolomic divergence between healthy and periodontitis samples.

### 3.3 Metabolomic Classes Observed in Healthy and Periodontitis Samples

**FIGURE 2:**
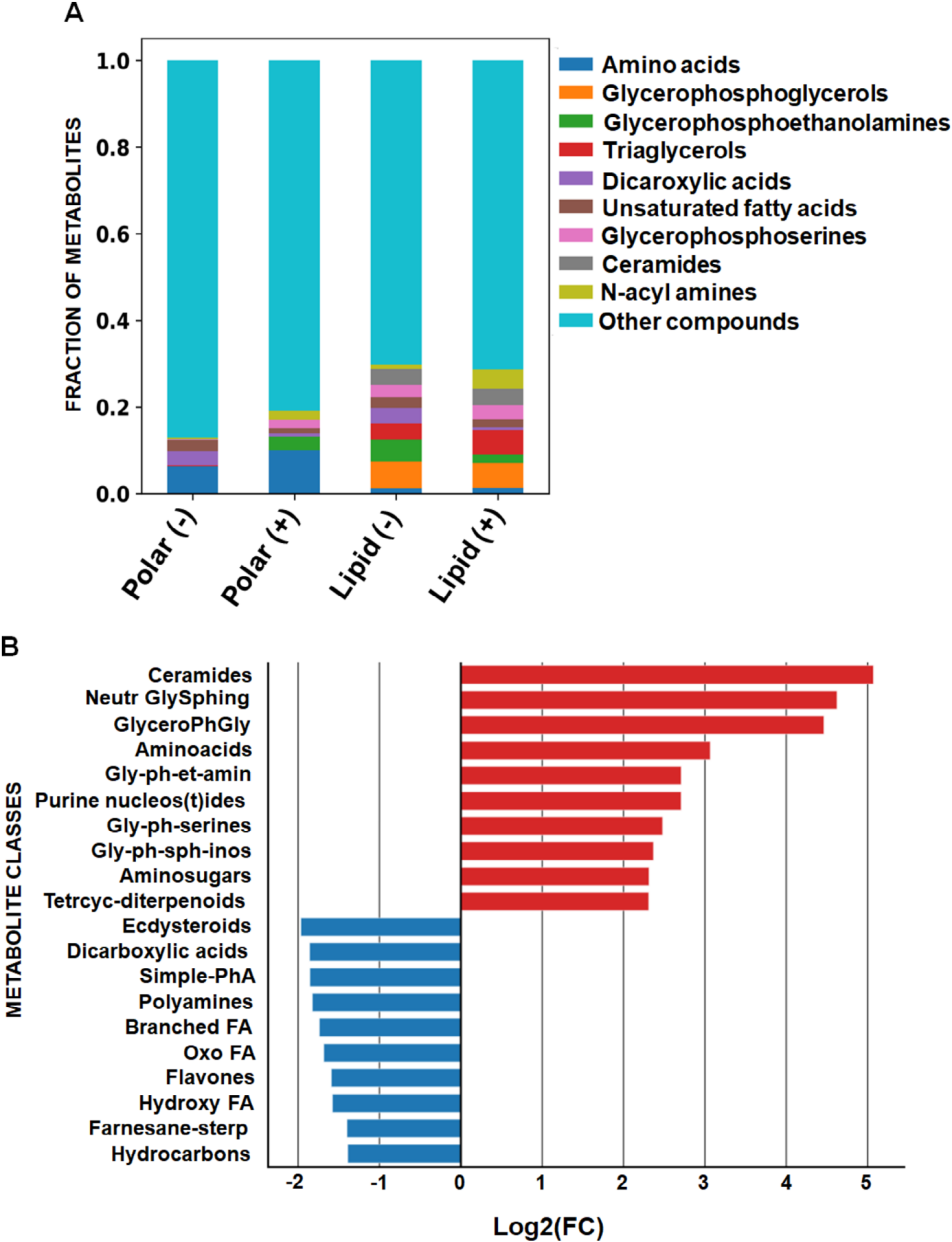
Polar metabolite and lipid class profiles differ in GCF from healthy versus periodontitis samples. (A) Chemical class composition identified as positively and negatively charged metabolites and lipids across samples in GCF. The top 9 metabolite compound classes are shown. (B) The top 10 upregulated (red) and downregulated (blue) metabolite classes detected in GCF of periodontitis samples. See Table S4 and Material and Methods in Supporting Information. Branched FA, branched fatty acids; GlyceroPhGly, glycerophosphoglycerols; Gly‐ph‐et‐amin, glycerophosphoethanolamines; Gly‐ph‐serines, Glycerophosphoserines; Gly‐ph‐sph‐inos, glycerophosphoinositols; Hydroxy FA, hydroxy fatty acids; Neutr GlySphing, neutral glycosphingolipids; Oxo FA, oxo fatty acid; Simple‐PhA, dsimple phenolic acids; Tetrcyc‐diterpenoids, tetracyclic diterpenoids.

Since metabolomic divergences in GCF were observed between healthy and periodontitis samples, we further aimed to identify the primary biochemical classes potentially involved in the disease. Among the detected biochemical classes, we observed that most metabolites were represented by amino acids, dicarboxylic acids, unsaturated fatty acids, glycerophosphoethanolamines, phosphoglycerols, ceramide sphingolipids and other compounds (Figure 2A). Next, we aimed to determine the metabolite classes linked with inflammatory destruction of the periodontium. We confidently identified 97 compound classes comprising negatively or positively charged metabolites and lipids in GCF samples (Table S4). Among detected compound classes, sphingolipids, for example, ceramides and neutral glycosphingolipids, followed by glycerophosphoglycerols, were abundantly prevalent in periodontitis GCF (Figure 2B; Table S4). In contrast, periodontitis samples were characterised by decreased levels of ecdysteroids, followed by dicarboxylic acids, simple phenolic acids, fatty acids and other compounds (Figure 2B; Table S4). Therefore, these data indicate a profound shift in the classes of metabolites with accelerated prevalence of sphingolipid classes between the GCF of healthy and diseased samples.

### 3.4 Detailed Characterisation of Metabolomic Signatures Between Healthy and Periodontitis

**FIGURE 3:**
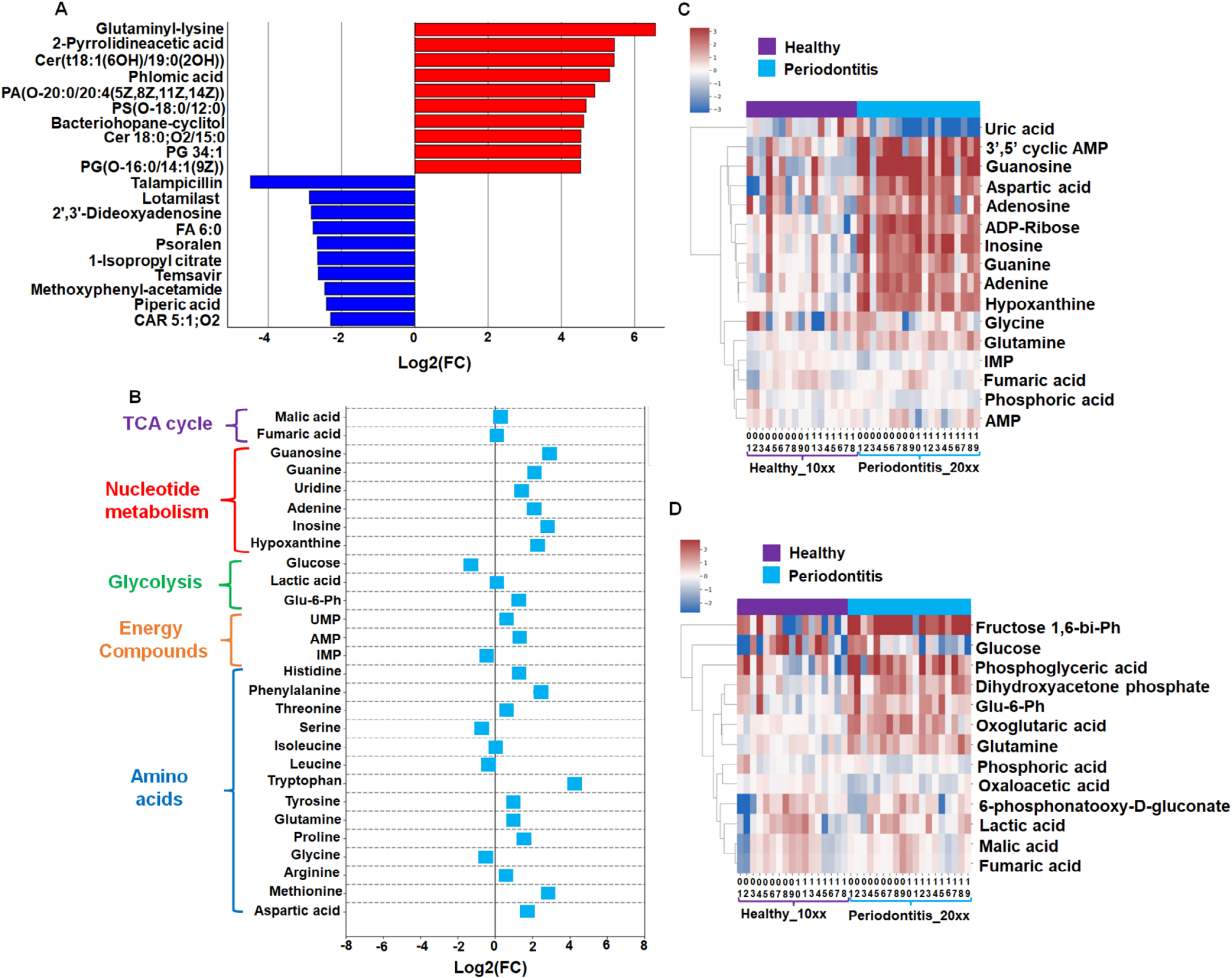
Differential metabolomic flux in GCF of healthy and periodontitis samples. (A) The top 10 metabolites that were upregulated (red) or downregulated (blue) in the GCF periodontitis samples. The parameters include MSI Level 1–3, *q* < 0.05 and |Log_2_ (FC)| > 1. (B) Effect of periodontitis on the accumulation of essential metabolites responsible for cell growth and respiration in GCF. (C) Prevalence of purine degradation metabolites, for example, aspartic acid and adenine, guanine, guanosine, hypoxanthine, inosine and uric acid in periodontitis and healthy samples. (D) Aerobic glycolysis, the Warburg effect, has no or a limited role in healthy and periodontitis samples. See Tables S5 and S6, and Material and Methods in Supporting Information. Bacteriohopane‐cyclitol, bacteriohopane‐31,32,33,34‐tetrol‐35‐cyclitol; Fructose 1,6‐bi‐Ph, fructose 1,6‐biphosphate; Glu‐6‐Ph, glucose‐6‐phosphate.

To better explore biochemical signatures in GCF between healthy and periodontitis samples, we included metabolites with MSI confidence Levels 1–3, excluding Level 4 metabolites and selected those with |Log_2_(FC)| > 1.0 and *q* < 0.05. Altogether, 198 metabolite and lipid compounds were significantly accelerated in the GCF of periodontitis samples compared with the healthy group (Table S5). Indeed, Figure 3A represents the top 10 most significantly accelerated compounds observed in the periodontitis GCF. Specifically, we observed accelerated levels of amino acids (glutaminyl‐lysine, 2‐pyrrolidineacetic acid), ceramide and dihydroceramide sphingolipids Cer(t18:1(6OH)/19:0(2OH)), (Cer 18:0;O2/15:0), unsaturated fatty acids (phlomic acid), glycerophosphates (PA(O‐20:0/20:4(5Z,8Z,11Z,14Z))), glycerophosphoglycerols (PG 34:1) and others. Of note, glutaminyl‐lysine was the most significantly accelerated metabolite observed in GCF periodontitis samples. In contrast, we observed a significant reduction of 58 compounds in GCF of periodontal lesions compared to healthy samples (Table S5). The top 10 significantly diminished metabolites and lipids in periodontal lesions include talampicillin, phosphodiesterase 4 inhibitors (lotamilast), purine nucleotides (2′,3′‐Dideoxyadenosine, CAR 5:1;O2), branched fatty acids (FA6:0), furocoumarins (psoralen), dicarboxylic acids (1‐Isopropyl citrate), O‐glutarylcarnitine (CAR 5:1;O2) and other classes (Figure 3A; Table S5).

To enhance our understanding of the cellular metabolic differences between healthy and diseased GCF, we further compared the levels of common metabolites involved in basic cellular function, including respiration, mitochondrial aerobic energy production, protein synthesis and nucleic acid synthesis (Ghosh et al. 2024). Altogether, we identified 28 metabolites at confident MSI Levels 1–2 (Table S6). Compared to the healthy individuals, GCF obtained from periodontitis samples demonstrated significant accumulation of compounds engaged in nucleotide metabolism (guanosine, guanine, uridine, adenine, inosine, hypoxanthine and adenosine diphosphate ribose), cellular energy uridine monophosphate (UMP) and amino acids (histidine, phenylalanine, tryptophan, methionine, proline, aspartic acid) (Figure 3B; Table S6). However, we observed little or no difference in the accumulation of metabolites involved in the TCA cycle (malic and fumaric acids), nor in glycolysis (glucose, lactic acid and glucose 6‐phosphate), as well as threonine, serine, isoleucine, leucine, tyrosine, glutamine, glycine and arginine amino acids between healthy and periodontitis samples (Figure 3B).

Clinical studies consistently report that patients with periodontitis exhibit diminished levels of cellular antioxidants, particularly uric acid (Barnes et al. 2009; Ye et al. 2023). Uric acid, a final product of purine degradation, serves as a potential biomarker for periodontitis (Barnes et al. 2009). Therefore, we aimed to understand whether uric acid and purine degradation intermediates are accelerated in periodontitis. We detected a significant reduction in uric acid levels in GCF from diseased samples (Figure 3C; Table S5). In contrast, a significant accumulation of its biochemical intermediates, for example, inosine, hypoxanthine, guanosine and guanine, was observed at the periodontitis GCF samples (Figure 3C). Notably, elevated accumulation of these purine degradation metabolites was also detected in Sample #1004, a healthy‐like sample obtained from a patient with a previous history of treated periodontitis (Table S1).

Given the tendency toward lower glucose levels observed in periodontal lesions, we next investigated metabolites involved in aerobic glycolysis, the Warburg effect, which typically leads to lactic acid accumulation (Bayley and Devilee 2012). We identified 13 potential metabolites associated with aerobic glycolysis (Figure 3D). Furthermore, six individual periodontitis samples demonstrate reduced glucose levels and accelerated lactic acid accumulation. It was impossible to distinguish whether lactic acid is produced by the host or bacterial species in periodontitis lesions. Therefore, aerobic glycolysis may play either a limited or no role in the pathology of periodontitis. In summary, our findings suggest that periodontitis primarily affects the metabolic signatures of sphingolipids, short‐chain fatty acids, amino acids and purines.

### 3.5 The Occurrence of Metabolomic Features Associated With Bacterial and Fungal Origin in GCF

Periodontitis pathogenesis involves a complex interplay between virulence factors produced by the oral microbiome and pro‐inflammatory immune responses (Bartold and Van Dyke 2013; Suresh Unniachan et al. 2020). We initially compared the levels of N‐acetylneuraminic acid and citrulline in diseased versus healthy sites, as these are some of the key biomarkers linked to periodontal bacterial metabolism in periodontitis (Barnes et al. 2009; Olsen et al. 2018; Rodrigues et al. 1997; Zhu et al. 2024). We observed a significant acceleration of citrulline, followed by N‐acetylneuraminic acid in GCF of periodontitis lesions (Table S5).

**FIGURE 4:**
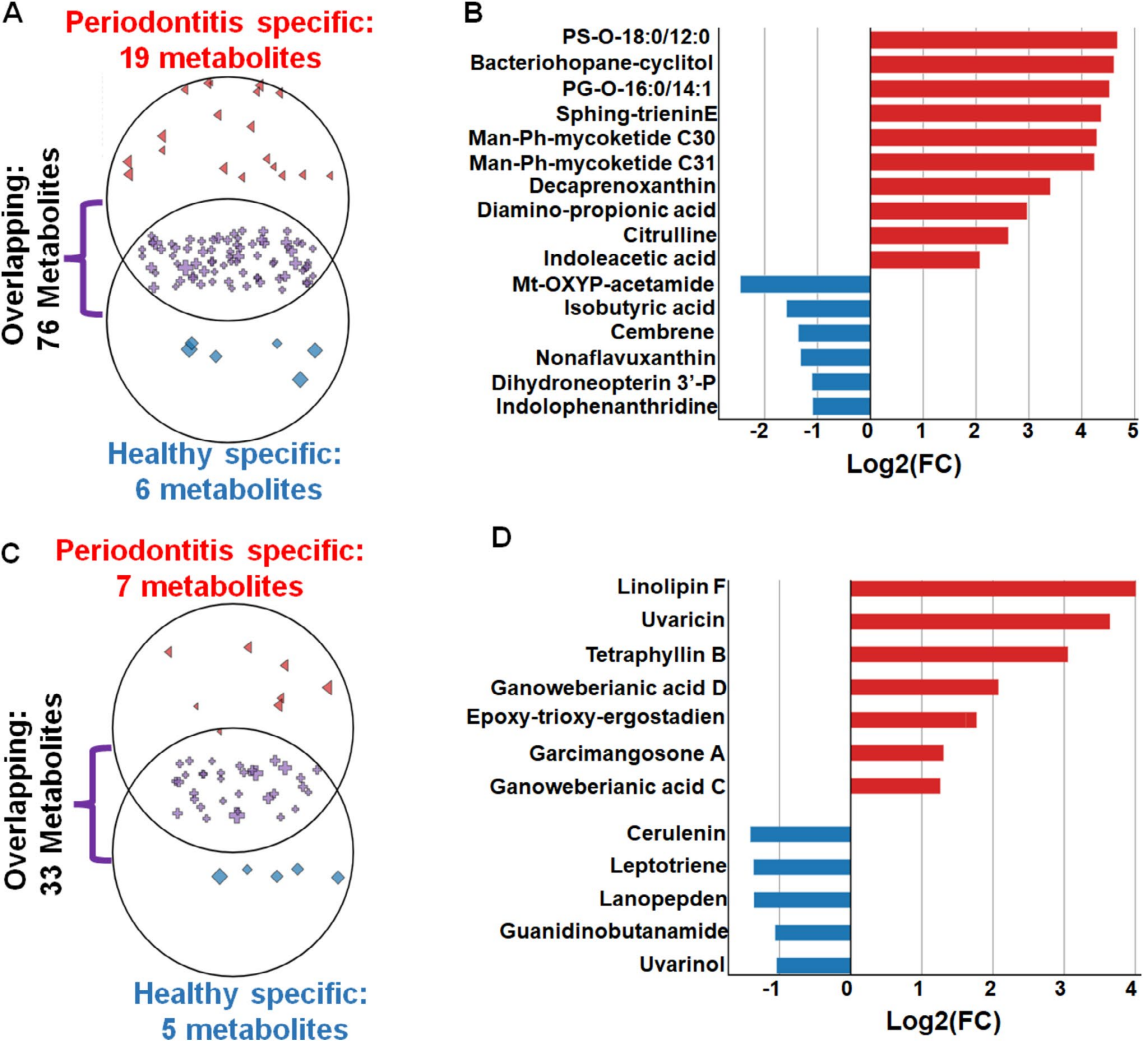
Metabolite levels associated with bacterial and fungal activities in GCF of healthy and periodontitis samples. (A) Distribution of metabolites associated with bacterial metabolism. (B) Occurrence of bacterial metabolites in periodontal (red) and healthy (blue) samples. (C) Distribution of metabolites associated with fungal metabolism in healthy and periodontitis samples. (D) Occurrence of fungal metabolites in periodontal (red) and healthy (blue) samples. The overlapping cycles between healthy and periodontitis‐specific cycles contain a group of nonsignificantly accelerated metabolites (cross symbol). The metabolite distance from the centre of overlapping cycles between healthy (diamond symbol) and periodontitis (triangle symbol) increases with the |Log_2_(FC)| > 1.0. The size of the metabolite symbol increases with the −Log_10_(p), where *p*‐value is adjusted with FDR. The metabolites confidence is represented by MSI Level 1–3. See Tables S7 and S8 and Material and Methods in Supporting Information. Bacteriohopane‐cyclitol, Bacteriohopane‐31,32,33,34‐tetrol‐35‐cyclitol; Diamino‐propionic acid, 2,3‐diamino‐propionic acid; Epoxy‐trioxy‐ergostadien, (22E,24R)‐5α,6α‐epoxy‐3β,7α,14β‐trihydroxy‐ergosta‐8,22‐dien‐15‐one; Guanidinobutanamide, 4‐Guanidinobutanamide; Man‐Ph‐mycoketide C30, Mannosyl‐1beta‐phosphomycoketide C30; Man‐Ph‐mycoketide C31, Mannosyl‐1beta‐phosphomycoketide C31; Mt‐OXYP‐acetamide, *N*‐(4‐Methoxyphenyl)acetamide; Dihydroneopterin 3′‐*P*, 7,8‐dihydroneopterin 3′‐phosphate; PG‐O‐16:0/14:1, PG(O‐16:0/14:1(9Z)); PS‐O‐18:0/12:0, PS(O‐18:0/12:0); Sphing‐trieninE, *N*‐(2R‐hydroxy‐heptadecanoyl)‐1‐beta‐D‐Glucosyl‐9‐methyl‐sphing‐4E,8E,10E‐trieninE.

Then, we aimed to identify the occurrence of biochemical substances associated with bacterial and fungal metabolomics across the GCF samples. Based on publicly available databases, we identified 101 bacterial and 45 fungal metabolites (Table S7 and S8). Specifically, 19 and 6 bacterial metabolites were significantly accelerated in periodontitis and healthy samples, respectively (Figure 4A; Table S7). Among them, the top metabolites were represented by glycerophosphoserines (PS(O‐18:0/12:0)), glycerophosphoglycerols (PG(O‐16:0/14:1(9Z))) and hopanes (bacteriohopane‐31,32,33,34‐tetrol‐35‐cyclitol) (Figure 4B). These metabolites also belong to the top 10 of most significantly accelerated metabolites in periodontitis lesions (Figure 2). Periodontal and healthy sites were also characterised by accelerated prevalence of seven and five fungal metabolites, respectively (Figure 4C,D). Altogether, these findings confirm the robustness of the untargeted metabolomic assay for characterising bacterial and fungal metabolomics in the healthy and diseased periodontium.

### 3.6 Purine Degradation and Ceramide Metabolism Are the Main Enrichment Pathways Engaged in Periodontitis

**FIGURE 5:**
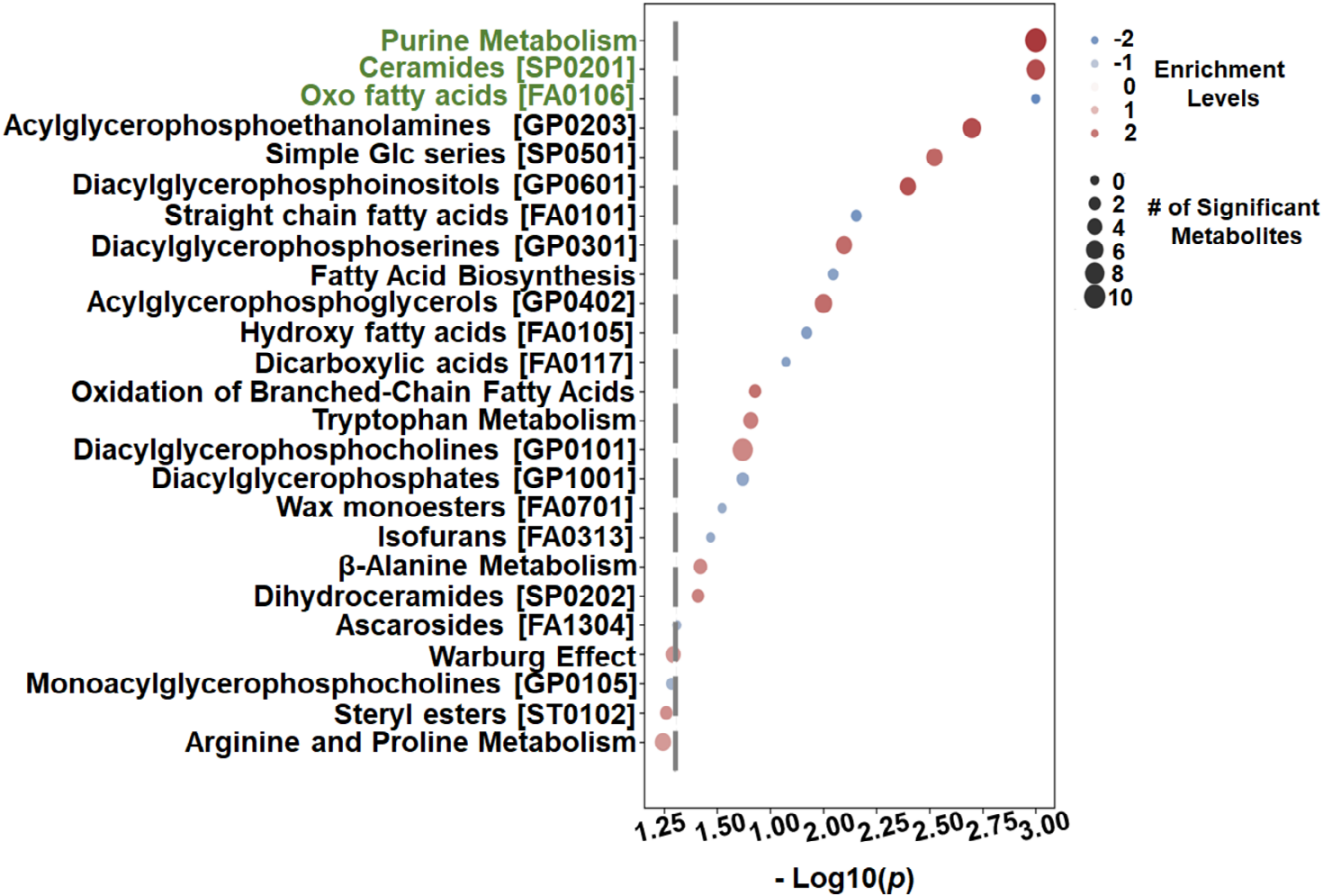
Periodontitis undergoes significant changes in the enrichment pathways engaged in purine degradation and ceramide sphingolipid accumulation. The top 21 metabolic pathways were ranked based on −Log_10_(p) > 1.3 as denoted by the grey dashed line. Among them, the purine, ceramide and oxo fatty acid pathways were significantly changed in periodontal lesions, with *q* < 0.05 (see Table S9). The dot plot denotes the enriched pathways with their corresponding significance levels represented on the x‐axis. The dot size reflects the number of statistically significant metabolites between healthy versus periodontitis samples. The dot colour represents positive, negative or uniformly distributed fold‐change enrichment. See Material and Methods in Supporting Information. Acylglycerophosphoethanolamines, 1‐(1Z‐alkenyl), 2‐acylglycerophosphoethanolamines [GP0203]; Simple Glc series, simple glycoceramides series.

To gain biological insight into the metabolic differences between the healthy and periodontitis groups, we finally performed quantitative enrichment analysis on altered metabolites in GCF. Altogether, 125 potential metabolic pathways were detected (Table S9). Based on the −Log10(*p*)‐value threshold, we detected significant alterations in 21 pathways (Figure 5; Table S9). Following adjustment for *q* < 0.05, we observed a positive acceleration of purine and ceramide metabolic pathways, along with a negative regulation of oxo fatty acid pathways in periodontitis lesions (Figure 5; Table S9), confirming results presented in Figures 2 and 3. Notably, we also confirmed that the Warburg effect pathway has no significant impact on the development of periodontitis. Collectively, these findings indicate that GCF obtained from periodontitis lesions exhibits metabolomic alterations associated with accelerated purine degradation and the accumulation of ceramide sphingolipids.

## 4 Discussion

Although the overall success rate for periodontitis treatment exceeds 85% in anterior teeth, an extensive clinical study reported that success drops significantly to 47% at molar teeth (Van der Weijden et al. 2019). This lower success rate may be linked, in part, to our incomplete understanding of the comprehensive metabolomic signatures between healthy periodontium and the disease stage (Alassy et al. 2025; Barnes et al. 2009).

To better understand the severity of periodontitis, several studies have shown that metabolite flux in GCF accurately reflects the disease status (Barnes et al. 2009; Fatima et al. 2021; Torres et al. 2024). Specifically, a meta‐analysis highlighted the accumulation of 40 metabolites, including amino acid and lipid degradation metabolites, in GCF samples from periodontitis patients (Baima et al. 2021). Using an untargeted metabolomic approach followed by a semisupervised deep learning‐based approach, we identified 256 metabolites with MSI confidence levels 1–3, including 198 elevated and 58 diminished compounds (*q* < 0.05 and |Log_2_FC| > 1) in GCF samples (Figure S1). Importantly, we further demonstrated a periodontitis‐like purine degradation metabolic profile in the GCF of a stable patient with a previous history of periodontitis treatment. We believe, therefore, our collection and data analysis strategies are crucial for identifying novel biomarkers of periodontitis.

Recent systematic reviews also highlighted the relatively limited clinical lipidomic studies comparing patients with periodontitis and healthy periodontium using untargeted LC/MS approaches (Silva et al. 2024; Zhou et al. 2022). Our metabolomic data screening revealed a dominant pattern of alterations in various lipids, including sphingolipids, glycerophospholipids and fatty acids in periodontal lesions. Notably, the observed acceleration of ceramides represents a significantly altered pathway in diseased samples, thereby supporting evidence regarding the pivotal role of sphingolipids in periodontitis pathology (Ghosh et al. 2024; Olsen and Nichols 2018; Sun et al. 2024). Due to current assay limitations, we were unable to confidently resolve the double bond positions within acyl chains in lipids. Therefore, further studies with targeted metabolomics are required.

We observed the positive accumulation of purine degradation metabolites, excluding uric acid, in periodontitis lesions. These data support a previously published clinical study (Barnes et al. 2009). Besides purine degradation metabolites, we also detected increased prevalences of 2‐pyrrolidoneacetic acid, N‐acetylneuraminic acid and citrulline in periodontitis GCF samples. Accelerated prevalence of N‐acetylneuraminic acid and citrulline in the diseased GCF aligns with previously published metabolomic studies (Barnes et al. 2009; Olsen et al. 2018; Rodrigues et al. 1997; Zhu et al. 2024). Indeed, 2‐pyrrolidineacetic acid was suggested as a potential biomarker for periodontitis in the smoking group of patients (Andorfer et al. 2021). Of note, the smoking status as a periodontitis covariate was ignored in the current studies, representing a significant data limitation.

Our study also demonstrated a tendency toward diminished glucose levels in periodontitis lesions, as well as accelerated levels of lactate and UMP. These metabolites are critical in the aerobic glycolysis/the Warburg effect (Bayley and Devilee 2012). Growing evidence suggests that aerobic glycolysis, leading to lactate production, may be linked to periodontitis (Celik and Kantarci 2021; Pascale et al. 2020). Overall, we detected no or limited impact of the Warburg effect metabolites on periodontitis. However, these findings require further studies because lactate is also created via an anaerobic metabolic pathway influenced by oral bacteria (Choi and Park 2025).

Unlike the saliva microorganism metabolome (Gardner et al. 2020; Tsuchida et al. 2024), bacterial‐ and fungal‐derived metabolomic signatures in GCF are sparsely studied. We detected higher levels of bacterial ether‐linked phospholipids, such as PS(O‐), PG(O‐) and bacteriohopane hopanoids, in periodontitis. This specific profile is consistent with microbial membrane leakage and biofilm extension into the periodontal pocket, coupled with intense local inflammation (Bertolini et al. 2022; Ryan et al. 2023).

Among the putative fungal‐derived metabolites, we identified accelerated accumulation of ganoweberianic acid in periodontitis lesions. In contrast, diminished levels of uvarinol were found in periodontal lesions. Given that uvarinol was recently proposed as an anti‐microbial agent (Agbebi et al. 2025), it is plausible that it may be used for the treatment or prevention of periodontitis. Due to the limited current understanding, further studies are necessary to fully elucidate the pathological implications of fungal‐derived metabolomic data in periodontitis.

Collectively, these cross‐sectional findings suggest that untargeted metabolomic assays provide insight into the development of novel therapeutic regimens targeting purine degradation and ceramide metabolism. However, it is essential to consider the study limitations and potential for bias, as our data simply compared metabolomic signatures between healthy and periodontitis samples. We and others have demonstrated the impact of gender, age and smoking on the severity of periodontitis (Eke et al. 2015; Pink et al. 2023; Yamada et al. 2024). Therefore, it is crucial to consider these covariates in conjunction with metabolomic signatures in GCF between different diagnoses, for example, health, gingivitis and various grades of periodontitis, in future studies.

## 5 Conclusions

Metabolomics in periodontal disease remains an underexplored field. Therefore, our study has established a unique database comprising 256 polar metabolite and lipid compounds with MSI confidence level 1–3. These metabolomic datasets may contribute to the development of artificial intelligence/machine learning‐based precision protocols for periodontitis treatment, as was previously emphasised elsewhere (Ahmed et al. 2021).

## Author Contributions

**Hawra AlQallaf and Alexandru Movila:** study design and resources; **Caroline H. Henderzahs, Chiaki Yamada, Alexandr Morozov, Thelmalane Yalartai, Vanchit John, Hawra AlQallaf, Alexandru Movila:** sample collection, data analysis, interpretation, drafted and critically revised the manuscript. All authors agreed to be accountable for all aspects of the work.

## Funding

This work was supported by Hevolution Foundation, HF‐GRO‐23‐1199172‐46; National Institute on Aging, R01AG064003, NIAR25AG060892; and U.S. Department of Veterans Affairs Award I21BX006307.

## Conflicts of Interest

The authors declare no conflicts of interest.

## Supporting information


**Table S1:** LC/MS sample identification corresponding to clinical periodontal diagnosis and smoking status.

**Table S2:** Classification of detected metabolites in GCF based on the Metabolomics Standards Initiative (MSI) scoring scheme, with values ranging from Level 1 to Level 4.

**Table S3:** The list of upregulated and downregulated metabolites, along with their MSI levels, in healthy and periodontitis samples.

**Table S4:** Significantly changed metabolite and lipid compound classes in the GCF of healthy and periodontitis samples.

**Table S5:** The list of significantly altered chemical compounds identified at MSI Levels 1–3 in GCF of periodontitis samples.

**Table S6:** Common metabolites of basic cellular functions.

**Table S7:** Compounds linked to bacterial metabolomic pathways.

**Table S8:** Compounds linked to fungal metabolomic pathways.

**Table S9:** The list of metabolites engaged in metabolic enrichment analysis. Details are summarised in Figure 5.

**Figure S1:** Interactive volcano plot demonstrating distribution of metabolites and lipids according to the MSI algorithm with confidence Levels 1–4 in GCF samples.

**Data S1:** Supplementary material and methods.
Provides details on (a) experimental design and study participants; (b) GCF sample collection and storage; (c) biochemical methods used for isolation and analysis of total metabolites and lipids by hydrophilic interaction liquid chromatography and reversed‐phase liquid chromatography assays; (d) data normalisation, and statistical analysis; (e) software used for data visualisation and presentation; (f) the list of supplementary references.

## Data Availability

The data supporting the findings of this study are available in Tables S2–S9. The raw LC/MS files are available from the corresponding author upon reasonable request.
