# Supplementary material for: Untargeted Metabolomic and Lipidomic Profiles of Gingival Crevicular Fluid in the Context of Periodontitis
Source: J Clin Periodontol. 2026 Feb 9;53(5):774–83. doi: 10.1111/jcpe.70105 (PMC13086549; doi:10.1111/jcpe.70105)
Supplement: Supplementary file 1 — Table S1: LC/MS sample identification corresponding to clinical periodontal diagnosis and smoking status. [file JCPE-53-774-s008.docx]

**Suppl Table 1.** LC/MS sample identification corresponding to clinical periodontal diagnosis and smoking status.

| **Sample Group** | **Smoking Status** | **Gender** | **LC/MS Sample Identification** |
| --- | --- | --- | --- |
| **Healthy Control** |  |  |  |
| 1. Localized Dental Biofilm-Induced Gingivitis on a Reduced Periodontium in a Non-periodontitis Patient | Never smoker | Male | 1001_Healthy |
| 1. Clinical Gingival Health on a Reduced Periodontium in a Non-Periodontitis Patient | Never smoker | Female | 1002_Healthy |
| 1. Clinical Gingival Health on an Intact Periodontium | Never smoker | Female | 1003_Healthy |
| 1. Localized Dental Biofilm-Induced Gingivitis on a Reduced Periodontium in a Previously Treated Periodontitis Patient | Never smoker | Male | 1004_Healthy |
| 1. Localized Dental Biofilm-Induced Gingivitis on an Intact Periodontium | Never smoker | Female | 1005_Healthy |
| 1. Clinical Gingival Health on a Reduced Periodontium in a Non-Periodontitis patient | Never smoker | Female | 1006_Healthy |
| 1. Localized Dental Biofilm-Induced Gingivitis on an Intact Periodontium | Never smoker | Male | 1007_Healthy |
| 1. Clinical Gingival Health on a Reduced Periodontium in a Non-Periodontitis patient | Never smoker | Female | 1008_Healthy |
| 1. Clinical Gingival Health on a Reduced Periodontium in a Non-Periodontitis patient | Never smoker | Male | 1009_Healthy |
| 1. Clinical Gingival Health on an Intact Periodontium | Never smoker | Female | 1010_Healthy |
| 1. Clinical Gingival Health on a Reduced Periodontium in a Non-Periodontitis patient | Never smoker | Female | 1011_Healthy |
| 1. Clinical Gingival Health on a Reduced Periodontium in a Non-Periodontitis patient | Never smoker | Male | 1013_Healthy |
| 1. Generalized Dental Biofilm-Induced Gingivitis on a Reduced Periodontium in a Non-Periodontitis patient | Never smoker | Male | 1014_Healthy |
| 1. Clinical Gingival Health on a Reduced Periodontium in a Non-Periodontitis Patient | Never smoker | Female | 1015_Healthy |
| 1. Clinical Gingival Health on a Reduced Periodontium in a Non-Periodontitis Patient | Never smoker | Female | 1016_Healthy |
| 1. Clinical Gingival Health on an Intact Periodontium | Never smoker | Female | 1017_Healthy |
| 1. Localized Dental Biofilm-Induced Gingivitis on a Reduced Periodontium in a Non-Periodontitis patient with Peri-implant Health #18, 19, 29, 30, 31 | Never smoker | Male | 1018_Healthy* |
| **Periodontitis** |  |  |  |
| 1. Generalized Stage III Grade A Periodontitis | Never smoker | Male | 2001_Periodontitis |
| 1. Generalized Stage IV Grade B Periodontitis | Previous smoker (Cessation >11 years) | Male | 2002_Periodontitis |
| 1. Generalized Stage III Grade B Periodontitis | Previous smoker (Cessation >11 years) | Female | 2003_Periodontitis |
| 1. Generalized Stage IV Grade C Periodontitis | Current smoker | Male | 2004_Periodontitis |
| 1. Localized Stage II Grade A Periodontitis | Never smoker | Female | 2005_Periodontitis |
| 1. Localized Stage III Grade A Periodontitis with Peri-implant mucositis #6, 11 and Peri-implantitis #3, 4, 14, 30 | Never smoker | Male | 2006_Periodontitis* |
| 1. Generalized Stage IV Grade C Periodontitis | Current smoker | Male | 2007_Periodontitis |
| 1. Generalized Stage IV Grade C Periodontitis | Previous smoker (Cessation >11 years) | Male | 2008_Periodontitis |
| 1. Generalized Stage IV Grade B Periodontitis with Peri-implantitis #13 | Previous smoker (Cessation >11 years) | Female | 2009_Periodontitis* |
| 1. Generalized Stage IV Grade B Periodontitis | Never smoker | Male | 2010_Periodontitis |
| 1. Generalized Stage IV Grade C Periodontitis | Current smoker | Male | 2011_Periodontitis |
| 1. Generalized Stage IV Grade C Periodontitis | Current smoker | Female | 2012_Periodontitis |
| 1. Generalized Stage IV Grade B Periodontitis | Current smoker | Female | 2013_Periodontitis |
| 1. Generalized Stage III Grade C Periodontitis | Current smoker | Female | 2014_Periodontitis |
| 1. Generalized Stage III Grade B Periodontitis | Current smoker** | Male | 2015_Periodontitis |
| 1. Localized Stage III Grade B Periodontitis | Current smoker | Female | 2016_Periodontitis |
| 1. Generalized Stage III Grade B Periodontitis | Never smoker | Female | 2017_Periodontitis |
| 1. Localized Stage III Grade B Periodontitis | Current smoker | Female | 2018_Periodontitis |
| 1. Generalized Stage III Grade C Periodontitis | Never smoker | Female | 2019_Periodontitis |

*Sample was obtained from a non-implant (natural tooth) site

** Marijuana Smoker (no Tobacco)
