# Supplementary material for: Untargeted Metabolomic and Lipidomic Profiles of Gingival Crevicular Fluid in the Context of Periodontitis
Source: J Clin Periodontol. 2026 Feb 9;53(5):774–83. doi: 10.1111/jcpe.70105 (PMC13086549; doi:10.1111/jcpe.70105)
Supplement: Supplementary file 6 — Table S6: Common metabolites of basic cellular functions. [file JCPE-53-774-s007.docx]

**Suppl. Table 6. Common metabolites of basic cellular functions**

| **Metabolite Name** | | **log2(FC)** | **p** | **q** | **MSI Level** |
| --- | --- | --- | --- | --- | --- |
| **TCA cycle** | | | | | |
|  | Malic acid* | 0.067955 | 4.08E-01 | 5.36E-01 | 1 |
|  | Fumaric acid* | 0.064994 | 4.56E-01 | 5.77E-01 | 2 |
| **Nucleotide metabolism** | | | | | |
|  | Inosine | 2.453832 | 5.27E-07 | 1.96E-05 | 1 |
|  | Guanosine | 2.207486 | 2.94E-05 | 3.27E-04 | 2 |
|  | Hypoxanthine | 2.028993 | 1.31E-08 | 2.02E-06 | 1 |
|  | Adenine | 1.663529 | 9.48E-06 | 1.41E-04 | 2 |
|  | Guanine | 1.566234 | 1.28E-05 | 1.70E-04 | 1 |
|  | Uridine | 1.334402 | 1.41E-07 | 7.83E-06 | 2 |
| **Glycolysis** | | | | | |
|  | Glucose | -1.153571 | 6.06E-01 | 7.16E-01 | 2 |
|  | Lactic acid * | 0.082583 | 3.61E-01 | 4.87E-01 | 1 |
|  | Glucose 6-phosphate * | 0.702674 | 4.28E-03 | 1.64E-02 | 2 |
| **Energy compounds** | | | | | |
|  | UMP | 1.100683 | 1.39E-04 | 1.08E-03 | 1 |
|  | IMP* | -0.303177 | 3.47E-02 | 8.26E-02 | 2 |
|  | AMP* | 0.204535 | 8.94E-01 | 9.33E-01 | 1 |
| **Amino Acids** | | | | | |
|  | Tryptophan | 3.921216 | 9.57E-12 | 9.97E-09 | 2 |
|  | Methionine | 2.700150 | 2.56E-08 | 3.45E-06 | 2 |
|  | Phenylalanine | 1.692717 | 1.45E-02 | 4.25E-02 | 2 |
|  | Proline | 1.433730 | 1.24E-06 | 3.50E-05 | 2 |
|  | Aspartic acid | 1.104720 | 9.74E-04 | 4.97E-03 | 2 |
|  | Histidine | 1.002413 | 6.14E-05 | 5.71E-04 | 2 |
|  | Glutamine* | 0.956363 | 1.63E-05 | 2.03E-04 | 2 |
|  | Tyrosine* | 0.910584 | 3.23E-02 | 7.85E-02 | 1 |
|  | Serine* | -0.647870 | 3.73E-02 | 8.62E-01 | 2 |
|  | Arginine* | 0.629555 | 2.08E-01 | 3.22E-01 | 2 |
|  | Threonine* | 0.400898 | 2.58E-01 | 3.82E-01 | 2 |
|  | Glycine* | -0.474262 | 7.30E-01 | 8.14E-01 | 2 |
|  | Leucine* | -0.280440 | 2.69E-03 | 1.13E-02 | 2 |
|  | Isoleucine* | -0.027332 | 9.83E-01 | 9.91E-01 | 2 |

* - non-significant
