# Supplementary material for: Untargeted Metabolomic and Lipidomic Profiles of Gingival Crevicular Fluid in the Context of Periodontitis
Source: J Clin Periodontol. 2026 Feb 9;53(5):774–83. doi: 10.1111/jcpe.70105 (PMC13086549; doi:10.1111/jcpe.70105)
Supplement: Supplementary file 7 — Table S7: Compounds linked to bacterial metabolomic pathways. [file JCPE-53-774-s001.docx]

**Supplementary Table 7. Compounds linked to bacterial metabolomic pathways**

|  | **Metabolite Name** | **log2(fc)** | **p** | **q** | **MSI Level** |
| --- | --- | --- | --- | --- | --- |
| **Significantly diminished metabolites in periodontitis individuals** | | | | | |
|  | N-(4-Methoxyphenyl)acetamide​ | -2.459628 | 1.15E-07 | 7.06E-06 | 3 |
|  | Isobutyric acid | -1.586522 | 4.13E-05 | 4.20E-04 | 3 |
|  | Cembrene (or isomer) | -1.362595 | 4.81E-07 | 1.86E-05 | 3 |
|  | Nonaflavuxanthin (or isomer) | -1.320174 | 6.06E-08 | 5.05E-06 | 3 |
|  | 7,8-dihydroneopterin 3'-phosphate (or isomer) | -1.103557 | 5.86E-03 | 2.10E-02 | 3 |
|  | Indolophenanthridine | -1.090833 | 1.29E-05 | 1.72E-04 | 3 |
| **Significantly accelerated metabolites in periodontitis individuals** | | | | | |
|  | PS(O-18:0/12:0) | 4.674455 | 1.23-E05 | 1.67-E04 | 3 |
|  | Bacteriohopane-31,32,33,34-tetrol-35-cyclitol (or isomer)​ | 4.608985 | 5.47E-05 | 5.26E-04 | 3 |
|  | PG(O-16:0/14:1(9Z) | 4.525119 | 5.90E-06 | 1.00E-04 | 3 |
|  | N-(2R-hydroxy-heptadecanoyl)-1-beta-D-Glucosyl-9-methyl-sphing-4E,8E,10E-trieninE (or isomer) | 4.368025 | 1.60E-04 | 1.20E-03 | 3 |
|  | Mannosyl-1beta-phosphomycoketide C30 (or isomer) | 4.284775 | 7.78E-04 | 4.20E-03 | 3 |
|  | Mannosyl-1beta-phosphomycoketide C31 (or isomer) | 4.241358 | 6.27E-05 | 5.78E-04 | 3 |
|  | Decaprenoxanthin (or isomer)​ | 3.407079 | 3.02E-05 | 3.31E-04 | 3 |
|  | 2,3-diamino-propionic acid​ | 2.967269 | 2.43E-05 | 2.85E-04 | 3 |
|  | Citrulline​ | 2.612476 | 1.98E-06 | 4.83E-05 | 2 |
|  | Indoleacetic acid​ | 2.072956 | 3.94E-03 | 1.53E-02 | 2 |
|  | 1-Hydroxy-2-naphthoate (or isomer)​ | 1.751108 | 2.62E-04 | 1.80E-03 | 3 |
|  | N-Methyl-aspartic acid​ | 1.643247 | 6.10E-07 | 2.18E-05 | 2 |
|  | d-Alanyl-l-alanine (or isomer)​ | 1.452851 | 7.99E-03 | 2.70E-02 | 3 |
|  | 13E,17-Octadecadienoic acid (or isomer)​ | 1.394863 | 7.96E-06 | 1.25E-04 | 3 |
|  | Topostin D654​ (or isomer) | 1.122104 | 6.27E-04 | 3.55E-03 | 3 |
|  | 3-deoxy-D-manno-octulosonate 8-phosphate​ | 1.170224 | 5.45E-07 | 2.01E-05 | 2 |
|  | (R)-Allantoin | 1.150053 | 4.39E-04 | 2.68E-03 | 2 |
|  | 2-C-methyl-d-erythritol-4-phosphate (or isomer) | 1.115002 | 3.42E-03 | 1.36E-02 | 3 |
|  | S-Isopropyl 3-methylbut-2-enethioate (or isomer) | 1.057622 | 6.52E-04 | 3.65E-03 | 3 |
| **No significant alteration between metabolites in healthy and periodontitis individuals** | | | | | |
|  | Dihydroxy-2H-indole glucuronide (or isomer) | -0.958329 | 2.91E-03 | 1.20E-02 | 3 |
|  | Phenolic phthiocerol (or isomer) | 0.931956 | 4.75E-04 | 2.86E-03 | 3 |
|  | UDP-galacturonic acid | 0.926801 | 6.48E-04 | 3.64E-03 | 1 |
|  | 2'-Hydroxyflexixanthin trimethyl ether (or isomer) | 0.925621 | 4.76E-03 | 1.78E-02 | 3 |
|  | Rhodobacterioxanthin (or isomer) | -0.763432 | 2.13E-02 | 5.67E-02 | 3 |
|  | Flavocristamide A (or isomer) | 0.738703 | 9.38E-02 | 1.74E-01 | 3 |
|  | Desferrioxamine G (or isomer) | -0.737746 | 2.57E-01 | 3.80E-01 | 3 |
|  | 1,1'-Dihydroxy-zeta-carotene (or isomer) | -0.712130 | 3.84E-04 | 2.44E-03 | 3 |
|  | 9,9-dimethoxy-nonanoic acid (or isomer) | -0.700890 | 8.41E-04 | 4.45E-03 | 3 |
|  | Aminoadipic acid | -0.669967 | 2.87E-01 | 4.15E-01 | 2 |
|  | OH-Diaponeurosporene glucoside ester (or isomer) | -0.664744 | 6.42E-08 | 5.05E-06 | 3 |
|  | 2-Amino-3-hydroxypropanoic acid (or isomer) | -0.647872 | 3.73E-02 | 8.63E-02 | 3 |
|  | Tridecylic acid (or isomer) | -0.610660 | 1.52E-04 | 1.16E-03 | 3 |
|  | N-(3-oxo-pentanoyl)-homoserine lactone (or isomer) | -0.585255 | 1.47E-02 | 4.30E-02 | 3 |
|  | 2R-hydroxy-pentadecanoic acid (or isomer) | 0.575738 | 3.02E-05 | 3.31E-04 | 3 |
|  | 5-aceto valeric acid (or isomer) | -0.529918 | 6.67E-03 | 2.32E-02 | 3 |
|  | Heneicoyslic acid (or isomer) | -0.526303 | 6.61E-05 | 6.00E-04 | 3 |
|  | 7-Hydroxy-5-heptynoic acid (or isomer) | 0.485856 | 7.84E-02 | 1.53E-01 | 3 |
|  | N-butanoyl-l-homoserine lactone (or isomer) | 0.477934 | 4.24E-04 | 2.62E-03 | 3 |
|  | Indoleacetaldehyde (or isomer) | -0.472101 | 2.41E-03 | 1.03E-02 | 3 |
|  | N-(3-oxo-octanoyl)-homoserine lactone (or isomer) | -0.462573 | 6.61E-02 | 1.34E-01 | 3 |
|  | Norvaline | -0.447123 | 6.24E-01 | 7.29E-01 | 2 |
|  | Bacillamidin D | -0.443769 | 8.95E-02 | 1.68E-01 | 3 |
|  | Myxol 2'-methyl hexoside (or isomer) | -0.436341 | 1.38E-01 | 2.33E-01 | 3 |
|  | 4-Hydroxy-4,4'-diaponeurosporene (or isomer) | -0.429570 | 3.84E-06 | 7.41E-05 | 3 |
|  | beta-D-glucosyl 4,4'-diaponeurosporenoic acid (or isomer) | -0.427025 | 4.20E-03 | 1.62E-02 | 3 |
|  | Ketospirilloxanthin (or isomer) | 0.419516 | 9.27E-02 | 1.72E-01 | 3 |
|  | 4,4'-Diapocaroten-4'-al-4-oic acid (or isomer) | 0.417848 | 8.32E-04 | 4.41E-03 | 3 |
|  | Rhodoquinone-9 (or isomer) | -0.393760 | 1.88E-02 | 5.14E-02 | 3 |
|  | 7-hydroxy-dodecanoic acid (or isomer) | -0.378807 | 3.56E-02 | 8.40E-02 | 3 |
|  | Trehalose | 0.374541 | 3.70E-02 | 8.60E-02 | 2 |
|  | 1-(2-methoxy-17Z-tetracosenyl)-sn-glycero-3-phosphoethanolamine (or isomer) | -0.367740 | 5.61E-03 | 2.02E-02 | 3 |
|  | N-(3-oxo-heptanoyl)-homoserine lactone (or isomer) | -0.361667 | 1.10E-01 | 1.95E-01 | 3 |
|  | Milbemycin beta2 (or isomer) | -0.356165 | 4.21E-04 | 2.61E-03 | 3 |
|  | N-(3-hydroxy-pentanoyl)-homoserine lactone (or isomer) | -0.347688 | 7.97E-02 | 1.54E-01 | 3 |
|  | N-(3-oxo-dodecanoyl)-homoserine thiolactone (or isomer) | -0.337429 | 2.29E-02 | 6.00E-02 | 3 |
|  | 12-hydroxyjasmonic acid (or isomer) | -0.326860 | 1.23E-02 | 3.75E-02 | 3 |
|  | N-3-Hydroxyoctanoyl-L-homoserine lactone (or isomer) | -0.324410 | 1.14E-02 | 3.54E-02 | 3 |
|  | 3-hydroxy-tridecanoic acid (or isomer) | -0.311912 | 1.09E-03 | 5.45E-03 | 3 |
|  | N-(3R-hydroxy-7Z-tetradecenoyl)-homoserine lactone (or isomer) | -0.305839 | 2.12E-02 | 5.67E-02 | 3 |
|  | 2S-amino-pentanoic acid (or isomer) | 0.304782 | 4.88E-01 | 6.07E-01 | 3 |
|  | 5-(L-alanin-3-yl)-2-hydroxy-cis,cis-muconate 6-semialdehyde (or isomer) | -0.303638 | 1.86E-02 | 5.08E-02 | 3 |
|  | Citronellyl isovalerate (or isomer) | -0.294293 | 2.53E-02 | 6.48E-02 | 3 |
|  | 6-heptynoic acid (or isomer) | -0.289310 | 9.97E-04 | 5.05E-03 | 3 |
|  | Dokdolipid A (or isomer) | -0.286790 | 3.77E-02 | 8.69E-02 | 3 |
|  | 1-(2-methoxy-eicosanyl)-sn-glycero-3-phosphoserine (or isomer) | 0.284783 | 9.16E-02 | 1.71E-01 | 3 |
|  | 1-(2-methoxy-heneicosanyl)-sn-glycero-3-phosphoethanolamine (or isomer) | -0.278783 | 3.35E-02 | 8.05E-02 | 3 |
|  | Tensyuic acid B (or isomer) | -0.270839 | 7.20E-02 | 1.43E-01 | 3 |
|  | Sulfobacin B | -0.256021 | 4.45E-01 | 5.68E-01 | 2 |
|  | Thiolutin (or isomer) | -0.253879 | 2.19E-02 | 5.78E-02 | 3 |
|  | Methylglutaric acid (or isomer) | -0.251043 | 1.29E-01 | 2.21E-01 | 3 |
|  | N-[3-(13-methyl-tetradecanoyloxy)-13-methyl-hexadecanoyl] glycyl serine (or isomer) | -0.235666 | 3.44E-01 | 4.71E-01 | 3 |
|  | Glutamic acid betaine (or isomer) | -0.221743 | 4.89E-02 | 1.06E-01 | 3 |
|  | (1R)-Glutathionyl-(2R)-hydroxy-1,2-dihydronaphthalene (or isomer) | -0.200215 | 6.12E-02 | 1.27E-01 | 3 |
|  | (S)-3-Methylthiohexyl butyrate (or isomer) | 0.199388 | 2.41E-01 | 3.62E-01 | 3 |
|  | Poststatin (or isomer) | -0.197511 | 4.80E-02 | 1.05E-01 | 3 |
|  | 1-(2-methoxy-octadecanyl)-sn-glycero-3-phosphoserine (or isomer) | -0.191251 | 1.04E-01 | 1.88E-01 | 3 |
|  | Linolipin A (or isomer) | -0.190473 | 1.50E-01 | 2.48E-01 | 3 |
|  | OH-Spheroidenone (or isomer) | 0.170545 | 3.31E-01 | 4.59E-01 | 3 |
|  | L-2-Aminoadipate adenylate (or isomer) | -0.151342 | 4.27E-01 | 5.52E-01 | 3 |
|  | 1-Hexosyl-1,2-dihydro-3,4-didehydroapo-8'-lycopenol (or isomer) | -0.145800 | 3.43E-01 | 4.71E-01 | 3 |
|  | 4'-Apo-3,4-didehydrolycopene/ (4-Apo-3',4'-didehydrolycopene) (or isomer) | 0.138794 | 2.55E-01 | 3.78E-01 | 3 |
|  | 6-[3]-Ladderane-1-hexanol (or isomer) | 0.119529 | 6.47E-01 | 7.47E-01 | 3 |
|  | N-(3-oxo-butanoyl)-homoserine lactone (or isomer) | -0.109493 | 1.73E-01 | 2.78E-01 | 3 |
|  | Avermectin A1a | -0.102489 | 2.04E-01 | 3.16E-01 | 2 |
|  | EI-1625-2 (or isomer) | -0.099720 | 3.35E-01 | 4.64E-01 | 3 |
|  | Beta-Cryptoxanthin laurate (or isomer) | 0.083439 | 5.67E-01 | 6.81E-01 | 3 |
|  | 4-carboxy-2-hydroxy-cis,cis-muconic acid (or isomer) | -0.081211 | 5.86E-01 | 6.99E-01 | 3 |
|  | Validamycin A | 0.073865761 | 0.630492454 | 0.734458509 | 3 |
|  | N-heptanoyl-homoserine lactone (or isomer) | 0.062237 | 4.98E-01 | 6.17E-01 | 3 |
|  | N-(13-methyl-tetradecanoyl)-capnine (or isomer) | 0.044240 | 7.04E-01 | 7.93E-01 | 3 |
|  | N-decanoyl histidine (or isomer) | 0.037373 | 5.50E-01 | 6.64E-01 | 3 |
|  | Propionic acid | -0.027589 | 9.28E-01 | 9.54E-01 | 2 |
|  | Tensyuic acid E (or isomer) | -0.020879 | 7.13E-01 | 8.00E-01 | 3 |
|  | (R)-b-amino-isobutyric acid (or isomer) | -0.014694 | 8.36E-01 | 8.90E-01 | 3 |
|  | Novobiocin (or isomer) | -0.004570 | 9.82E-01 | 9.91E-01 | 3 |
