# Supplementary material for: Untargeted Metabolomic and Lipidomic Profiles of Gingival Crevicular Fluid in the Context of Periodontitis
Source: J Clin Periodontol. 2026 Feb 9;53(5):774–83. doi: 10.1111/jcpe.70105 (PMC13086549; doi:10.1111/jcpe.70105)
Supplement: Supplementary file 8 — Table S8: Compounds linked to fungal metabolomic pathways. [file JCPE-53-774-s006.docx]

**Supplementary Table 8. Compounds linked to fungal metabolomic pathways**

|  | **Metabolite Name** | **log2(fc)** | **p** | **q** | **MSI Level** |
| --- | --- | --- | --- | --- | --- |
| **Significantly diminished metabolites in periodontitis individuals** | | | | | |
|  | Cerulenin (or isomer) | -1.408424 | 4.83E-05 | 4.80E-04 | 3 |
|  | Leptotriene (or isomer) | -1.962063 | 7.04E-06 | 1.14E-04 | 3 |
|  | Lanopepden (or isomer) | -1.358233 | 2.17E-04 | 1.55E-03 | 3 |
|  | 4-Guanidinobutanamide (or isomer) | -1.060161 | 8.13E-03 | 2.72E-02 | 3 |
|  | Uvarinol (or isomer) | -1.039810 | 2.54E-04 | 1.76E-03 | 3 |
| **Significantly accelerated metabolites in periodontitis individuals** | | | | | |
|  | Linolipin F (or isomer) | 4.007140 | 5.14E-05 | 5.01E-04 | 3 |
|  | Uvaricin (or isomer) | 3.643021 | 1.55E-04 | 1.17E-03 | 3 |
|  | Tetraphyllin B sulfate (or isomer) | 3.052282 | 2.89E-05 | 3.24E-04 | 3 |
|  | Ganoweberianic acid D (or isomer) | 2.075099 | 2.83E-07 | 1.34E-05 | 3 |
|  | (22E,24R)-5α,6α-epoxy-3β,7α,14β-trihydroxy-ergosta-8,22-dien-15-one (or isomer) | 1.617226 | 4.67E-04 | 2.82E-03 | 3 |
|  | Garcimangosone A (or isomer) | 1.304481 | 5.33E-06 | 9.38E-05 | 3 |
|  | Ganoweberianic acid C (or isomer) | 1.259028 | 3.49E-01 | 4.76E-01 | 3 |
| **No significant alteration between metabolites in healthy and periodontitis individuals** | | | | | |
|  | Aspergillomarasmine A (or isomer) | -0.994920 | 8.07E-07 | 2.60E-05 | 3 |
|  | 4-Guanidinobutanoic acid | 0.944032 | 2.45E-01 | 3.66E-01 | 1 |
|  | Stachyflin (or isomer) | -0.633178 | 8.44E-06 | 1.30E-04 | 3 |
|  | Pulcherriminic acid (or isomer) | -0.554739 | 2.44E-02 | 6.30E-02 | 3 |
|  | Lucidone B (or isomer) | -0.554677 | 1.34E-07 | 7.67E-06 | 3 |
|  | Curvulalide (or isomer) | -0.426172 | 1.74E-03 | 7.93E-03 | 3 |
|  | campest-22E-en-3β,4β,5α,6α,8 β,14α,15α,25R,26-nonol (or isomer) | 0.414866 | 6.09E-02 | 1.27E-01 | 3 |
|  | Stoloniferone O (or isomer) | 0.408626 | 5.73E-02 | 1.21E-01 | 3 |
|  | Ganoderic acid Ma (or isomer) | -0.397220 | 3.50E-03 | 1.39E-02 | 3 |
|  | Conicasterol B (or isomer) | -0.389167 | 2.58E-02 | 6.59E-02 | 3 |
|  | Hericenone G (or isomer) | -0.368299 | 5.22E-04 | 3.08E-03 | 3 |
|  | Lecanoric acid (or isomer) | -0.329646 | 1.62E-01 | 2.63E-01 | 3 |
|  | N-jasmonoyl-dopamine (or isomer) | -0.328349 | 5.70E-02 | 1.20E-01 | 3 |
|  | Modiolide A (or isomer) | -0.320625 | 1.02E-02 | 3.21E-02 | 3 |
|  | Asperitaconic acid C (or isomer) | -0.286953 | 1.69E-02 | 4.72E-02 | 3 |
|  | Tensyuic acid B (or isomer) | -0.270839 | 7.20E-02 | 1.43E-01 | 3 |
|  | Scyphostatin (or isomer) | -0.238172 | 9.27E-03 | 3.01E-02 | 3 |
|  | Ethyl 18-bromooctadec-17-en-5,7,15-triynoate (or isomer) | -0.228811 | 1.81E-01 | 2.88E-01 | 3 |
|  | 9-Hydroxy-1-(2,4,6-trihydroxyphenyl)-6Z,10E,12Z,15Z-octadecatetraen-1-one (or isomer) | -0.216150 | 2.23E-02 | 5.86E-02 | 3 |
|  | Ustiloxin D (or isomer) | -0.208187 | 5.14E-03 | 1.89E-02 | 3 |
|  | Aflatoxin GM1 (or isomer) | -0.203161 | 2.38E-01 | 3.59E-01 | 3 |
|  | Antcin K (or isomer) | -0.201961 | 4.98E-02 | 1.08E-01 | 3 |
|  | Linolipin A (or isomer) | -0.190473 | 1.50E-01 | 2.48E-01 | 3 |
|  | Gibberellin A45 (or isomer) | -0.149578 | 1.04E-01 | 1.88E-01 | 3 |
|  | Fusarin C (or isomer) | -0.140763 | 1.92E-01 | 3.02E-01 | 3 |
|  | Ehrensteroid D (or isomer) | -0.120493 | 5.06E-01 | 6.24E-01 | 3 |
|  | Termitomycesphin B (or isomer) | -0.098712 | 7.06E-01 | 7.94E-01 | 3 |
|  | Gibberellin A32 (or isomer) | 0.080995 | 1.98E-01 | 3.09E-01 | 3 |
|  | Aflatoxin B1exo-8,9-epoxide-GSH (or isomer) | -0.076137 | 7.77E-01 | 8.48E-01 | 3 |
|  | Validamycin (or isomer) | 0.073866 | 6.30E-01 | 7.34E-01 | 3 |
|  | Gallicynoic acid A (or isomer) | -0.049810 | 7.82E-01 | 8.52E-01 | 3 |
|  | Patulin (or isomer) | -0.039115 | 9.12E-01 | 9.44E-01 | 3 |
|  | Tensyuic acid E (or isomer) | -0.020879 | 7.13E-01 | 8.00E-01 | 3 |
