# Supplementary material for: Untargeted Metabolomic and Lipidomic Profiles of Gingival Crevicular Fluid in the Context of Periodontitis
Source: J Clin Periodontol. 2026 Feb 9;53(5):774–83. doi: 10.1111/jcpe.70105 (PMC13086549; doi:10.1111/jcpe.70105)
Supplement: Supplementary file 11 — Data S1: Supplementary material and methods. Provides details on (a) experimental design and study participants; (b) GCF sample collection and storage; (c) biochemical methods used for isolation and analysis of total metabolites and lipids by hydrophilic interaction liquid chromatography and reversed‐phase liquid chromatography assays; (d) data normalisation, and statistical analysis; (e) software used for data visualisation and presentation; (f) the list of supplementary references. [file JCPE-53-774-s011.docx]

**Supplementary Material and Methods**

*Experimental Design and Participants*

This study was approved by the Institutional Review Board of Indiana University at Indianapolis (IRB #19010). Participant recruitment occurred from December 2023 to August 2024, where a signed informed consent was obtained from willing patients prior to enrollment. Participants included patients attending Indiana University School of Dentistry (IUSD) predoctoral and graduate clinics. Inclusion criteria consisted of patients who were 18-years or older, systemically healthy (ASA Class I or II), and were either periodontally healthy with no radiographic evidence of bone loss (labeled as healthy controls) or diagnosed with periodontitis. The initial periodontal diagnosis was performed by a dental student using the 2017 World Workshop guidelines (Chapple et al., 2018), and was then confirmed by a periodontal resident and a board-certified periodontist. The main parameters for the healthy control group included the PDs <4mm with no associated BOP and no interproximal attachment loss. In contrast, the control periodontitis group included PDs >4mm with associated BOP, radiographic bone loss (RBL) of more than 15% and interproximal attachment loss of more than 2 mm (Table 1). Of note, samples from patients diagnosed with gingivitis were obtained from the healthy site, specifically, the shallowest PD with no associated BOP, to reduce the influence of gingivitis. Subject demographic information, e.g, age, gender, and smoking status, were also recorded from the Axium database (Deltek© Herndon) at the time of sample collection. Participants were excluded if they had a history of uncontrolled diabetes mellitus type II (HbA1c >7% or >53mmol/mol), local radiotherapy to the head and neck, periodontal therapy within the last 3 months, pregnancy, or if antibiotics or anti-inflammatory steroidal medications were taken within 6 months of study initiation.

*GCF Sample Collection and Storage*

After a patient’s consent was obtained, GCF samples were collected for untargeted metabolomic analysis using an established protocol with minor modifications (Barnes et al., 2009).

Briefly, after isolating and drying the cotton with an air-water syringe, two blood- and saliva-free GCF samples were collected from the same intrasulcular site using absorbent points (Dentsply Sirona). The collection time was 1 minute per sample. Collected GCF samples were then pooled into one microcentrifuge tube containing 120 µL of phosphate-buffered saline (PBS) with protease inhibitor cocktail (ThermoFisher Scientific) and immediately stored at -80 °C until further processing. Of note, samples from patients identified as healthy controls were obtained from the shallowest PD with no associated BOP. The periodontitis GCF samples were obtained from the site with the greatest PD. In the course, when multiple sites had similar PD values, we obtained GCF samples from the site with the highest levels of CAL and RBL, indicating accelerated inflammation (Taba, Kinney, Kim, & Giannobile, 2005).

*Total Metabolites and Lipids Extraction*

GCF samples were profiled with untargeted metabolomics leveraging Panome Bio’s Next-Generation Metabolomics technology (St. Louis, MO). Method blanks, as well as pooled quality control samples, were also prepared and included in the downstream analysis, as described elsewhere (Stancliffe et al., 2022).

To isolate total metabolites and lipids, GCF samples were removed from the -80^o^C freezer, thawed on ice, and vortexed. Initially, protein concentration was measured in each collected sample using the BCA Protein Assay Kit according to the manufacturer’s recommendations (ThermoFisher). Then, 1000μl of a solution containing methanol: acetonitrile: water in a proportion of 2:2:1 (v:v:v) was applied, and the samples were immediately vortexed for 30 seconds, followed by placement in a liquid nitrogen bath for 1 minute. These steps were repeated three times. After 1 hour of incubation at -20°C, the samples were centrifuged at 14,000 RPM for 10 minutes at 4°C and the collected supernatant was naturally dried. To the residue dried samples, we applied 2:1 acetonitrile: water solution at a concentration of 1μl/2mg of protein. Next, samples were sonicated at 25°C for 5 minutes and vortexed twice. Then, samples were stored at 4°C for 60 minutes and centrifuged at 14,000 RPM for 10 minutes at 4°C. The supernatant was then transferred to a liquid chromatography vial and stored at -80°C until further processing.

*Analysis of Polar Metabolites by* Hydrophilic Interaction Liquid Chromatography/MS

The LC/MS mobile phases A and B were prepared as follows: A) 20mM ammonium bicarbonate, 0.1% ammonium hydroxide, 5% acetonitrile, 2.5mM medronic acid (pH=9.2), and B) 20mM ammonium bicarbonate, 0.1% ammonium hydroxide, 95% acetonitrile, 2.5mM medronic acid.

A 4μL aliquot of polar metabolite extract was analyzed with Hydrophilic Interaction Liquid Chromatography/MS on an Agilent 6546 QTOF coupled to an Agilent 1290 Infinity II LC system by using the following linear gradient at a flow rate of 250μL/min: 0-1 min: 90% B, 1-12 min: 90-35% B, 12-12.5 min: 35-20% B, 12.5min-14.5 min: 20% B. The column was re-equilibrated with 20 column volumes of 90% B. Mass spectrometry analysis was completed with a mass range of 67-1500Da with 1 scan/second in both positive and negative ionization mode. MS/MS data was acquired in a data-dependent iterative fashion with a 1.3m/z isolation window.

*Characterization of Lipid Metabolites by* Reversed-Phase Liquid Chromatography

The LC/MS mobile phases A and B were prepared as follows: A) 5:3:2 water:acetonitrile:isopropyl alcohol, 10 mM ammonium formate, and 5 μM Agilent deactivator additive and B) 1:9:90 water: acetonitrile: isopropyl alcohol and 10 mM ammonium formate.

A 4 μL aliquot of lipid metabolite extract was analyzed with Reversed-Phase Liquid Chromatography/MS (Waters Acquity Premier HSS T3, 2.1 x 100 mm) on an Agilent 6546 QTOF coupled to an Agilent 1290 Infinity II LC system by using the following linear gradient at a flow rate of 400 μL/min: 0-2.5 min: 15-50% B, 2.5-2.6 min: 50-57% B, 2.6-9 min: 57-70% B, 9.0-9.1 min: 70-93% B, 9.1-11 min: 93-96% B, 11.0-11.1 min: 96-100% B, 11.1-12 min: 100% B, 12.0-12.2 min: 100-15% B. The column was re-equilibrated for 3.8 minutes. Mass spectrometry analysis was performed with a mass range of 100-1700 m/z, at a scan rate of 1 scan per second/sec in both positive and negative modes. The MS/MS data were acquired in a data-dependent iterative fashion with a 1.3 m/z isolation window. Of note, we were unable to confidently determine the location of double bonds within the acyl chains in lipid metabolites.

*Metabolite Detection*

An initial unbiased LC/MS metabolomic and lipidomic detection of metabolite signals (or features) was performed using a semisupervised deep learning-based PeakDetective approach implemented in Python 3.10 (Stancliffe & Patti, 2023). Briefly, we run an unsupervised autoencoding convolutional neural network that processes millions of extracted ion chromatogram (regions of the raw data, represented as a 1x60 vector) to perform non-linear dimensionality reduction. The encoder, which uses two 1D convolutional layers, compresses the complex peak shape into a concise five-element latent representation. This vector is then fed into a small, fully connected feed-forward classifier with two hidden layers of five nodes each. Of note, the classifier uses a softmax machine-learning output layer to make the final determination of whether a metabolite signal is present at a particular region of the raw data. The classifier was trained using active learning and tuned to maximize the true positive rate while minimizing the false positive rate. Then, detected metabolite features were aligned across the samples to account for retention-time shifts. We have excluded features with blank/quality control (QC) ration > 0.33, because of possible contaminations (Cho et al., 2021). Feature degeneracy (isotopes, adducts, fragments, etc.) was identified through clustering and ion assignment with mz/unity.

*Metabolite Identification*

Once metabolic compounds passed QC, the chemical structures were identified by searching databases of known metabolites (RefMet, LipidMaps v2024, and Human Metabolome Databases v5.0) and comparing isotope patterns and MS/MS fragmentation data with Panome Bio’s DecoID algorithm. Because there is limited knowledge of bacterial and fungal metabolites associated with periodontitis, we also compared our results with publicly available databases, such as the Natural Products Atlas, KEGG Pathway, DrugBank, PubChem, Cayman Chemical, Biosynth, Carotenoids Database, GNPS spectral library, and supplementary peer-reviewed reports (Abdel-Mawgoud, Lépine, & Déziel, 2010; Achudhan et al., 2021; Agbebi et al., 2025; Anburajan, Meena, Vinithkumar, & Dharani, 2021; Angelini et al., 2023; Bryskier, 2005; Chaban, Nielsen, Kopec, & Khandelia, 2014; Chang et al., 2020; Chinthanom, Vichai, Rachtawee, Boonpratuang, & Isaka, 2023; Chowdhury et al., 2021; Chrzanowski, Ławniczak, & Czaczyk, 2012; Danaher, Howells, Crooks, Cerkvenik-Flajs, & O'Keeffe, 2006; Daniel, Friess, Rajagopalan, Wendt, & Zenobi, 2002; J. Ding et al., 2025; L. Ding, Li, Liao, He, & Xu, 2018; Eng et al., 2021; Fazius, Shelest, Gebhardt, & Brock, 2012; Flentie, Stallings, Turk, Minnaard, & Hsu, 2016; Gu et al., 2023; Hohmann et al., 2024; Hsieh & Ju, 2018; Huang, Alimova, Myers, & Ebersole, 2011; Hunter, 2007; Ishihara et al., 2025; Kawai, Yano, & Kaneda, 1988; Kawaide, 2006; Kim & Oh, 2013; Kobayashi et al., 2021; Koilybayeva et al., 2023; Lee, Kim, Khadke, & Lee, 2021; Li, Zhang, Hu, & Zhao, 2021; Ma et al., 2024; Mansour, Ali, Hassan, Gabra, & Mawad, 2025; Matsumaru et al., 2008; Meguro, Tomita, Nishiyama, & Kuzuyama, 2013; Miersch, Schneider, & Sembdner, 1991; Moon, Herr, Kim, & Lee, 2011; Nara et al., 1999; Nemoto, Ojika, Takahata, Andoh, & Sakagami, 1998; Ngoc et al., 2021; Noguchi et al., 2022; Omura, 1981; Oshkin et al., 2023; Parsons, Harris, & Patten, 2015; Phimister, Lee, Morin, Buckpitt, & Plopper, 2004; Raetz & Whitfield, 2002; Reijneveld et al., 2021; Roser et al., 2022; Ryan, Joyce, & Clarke, 2023; Sekowska, Ashida, & Danchin, 2019; Sepe et al., 2012; Shi et al., 2024; Shrestha, Karmacharya, Han, Lee, & Oh, 2024; Takaichi & Shimada, 1999; Tempesta, Kriek, & Bates, 1982; Thai, Stubbs, Sarkar-Tyson, & Kahler, 2023; Tørring, Shames, Cho, Roy, & Crawford, 2017; Xu, Andi, Qian, West, & Cook, 2006; Zhao, Guo, Shan, Chu, & Zhang, 2024; Zheng et al., 2025; Zhou, Kim, & Zhong, 2014){Abdel-Mawgoud, 2010 #95}.

Metabolite identifications were classified based on the Metabolomics Standards Initiative (MSI) scoring scheme, with values ranging from Level 1 to Level 4 (Stancliffe, Schwaiger-Haber, Sindelar, & Patti, 2021). More specifically, the Level 1 identifications are supported by experimental retention times, MS/MS spectra from authentic standards, and an isotope pattern match. Level 2 identifications were made based on isotope pattern matches of greater than >90% reverse dot-product and an MS/MS match of > 50% entropy similarity, along with a predicted retention time match of less than 2 minutes. Level 3 identifications were made based on an isotope pattern and a predicted retention time match, using the same cutoffs as defined above. Level 4 identifications are signals determined to correspond to a unique m/z feature without a confident match in a publicly available database. For lipid compounds, the best-matching lipid species at the MSI Level 1-3 is reported. Only MSI Levels 1 - 3 were used for statistical analysis and pathway identification. Of note, metabolite identifications and intensities were manually reviewed for concordance and accuracy. For this analysis, PathBank pathways were used as the pathway database, and LipidMaps was used for lipid class assignment (Conroy et al., 2024; Wishart et al., 2020).

*Data Normalization and Curation*

Metabolomics data from all assays were concatenated. Metabolite signals were discarded if the coefficient of variation (CV) amongst the quality control samples was greater than 10%. Missing values were imputed by using half of the minimum detected intensity for each metabolite and the data matrix was subsequently log₂-transformed to approximate normality prior to hypothesis testing. To harmonize annotation confidence, metabolites were assigned to Metabolomics Standards Initiative (MSI) levels (1–4) and standardized across assays.

*Statistical Analysis*

The Log2-transformed with median-normalized metabolite intensities were used for null hypothesis testing. Fold changes were computed from non-Log2-transformed intensities. To capture global variance structure and group separation between healthy and periodontitis samples, principal component analysis (PCA) was performed with the *scikit-learn* machine learning package available in Python. Samples were mean-centered and scaled to unit variance before decomposition. The analysis was repeated for metabolites within MSI confidence subsets (MSI 1–2, MSI 1–3, MSI 1–4) to verify the robustness of clustering patterns.

To perform pathway analysis, metabolites were ranked by fold-change direction (most positive to most negative). A rank-based enrichment analysis, conceptually similar to gene-set enrichment analysis (GSEA) (Subramanian et al., 2005), was applied to assess overrepresentation of metabolic pathways using *PathBank* and *LipidMaps* ontologies (Conroy et al., 2024; Wishart et al., 2020). Enrichment significance was estimated by 1,000 permutations of metabolite–pathway assignments to derive empirical null distributions. Additionally, over-representation analysis (ORA) via Fisher’s exact test quantified whether the number of significant metabolites per pathway exceeded chance expectation. Enrichment difference plots were generated by calculating the mean enrichment in periodontitis compared to that in healthy controls, followed by visualization of the top ten up- and down-regulated classes. The statistical significance of each pathway enrichment score was estimated based on the observed distribution of ranked metabolites within each pathway, without random permutation. Enrichment magnitude was derived from the difference between mean enrichment scores in periodontitis and healthy groups, providing a direct measure of pathway-level deregulation. A one-way ANOVA was employed to identify significant metabolite alterations between groups. The over-representation analysis was performed using Fisher’s Exact test, which compares the expected number of metabolites in each pathway that are statistically significant with the observed number.

Statistical significance was defined as a *p*-value < 0.05. The resulting p-values were corrected for multiple testing using the Benjamini–Hochberg false discovery rate (FDR) procedure, as implemented in statsmodels (Python), to generate q-values. Significance thresholds were set to q < 0.05 and |Log₂ fold change (FC)| > 1.0. These parameters were used throughout subsequent visualization steps, including volcano plots and MSI-stratified bar charts visualitasion based on the 4,168 features.

Data Visualization

Data visualization pipelines were unified using the open-source Plotly graphing library for Python (https://plotly.com/python/) to ensure full reproducibility, transparency, and interactivity. All figures were exported as standalone .html containers, allowing dynamic zooming, hovering, and layer toggling without additional dependencies. To maintain consistency across plots, identical interactive parameters (font size, color palette, gridline thickness, hover templates, and axis scaling) were applied throughout all visualization scripts, ensuring direct cross-figure comparability. {Abdel-Mawgoud, 2010 #95@@hidden}

**Supplementary References**

Abdel-Mawgoud, A. M., Lépine, F., & Déziel, E. (2010). Rhamnolipids: diversity of structures, microbial origins and roles. *Appl Microbiol Biotechnol, 86*(5), 1323-1336. doi:10.1007/s00253-010-2498-2

Achudhan, D., Liu, S. C., Lin, Y. Y., Huang, C. C., Tsai, C. H., Ko, C. Y., . . . Tang, C. H. (2021). Antcin K inhibits TNF-α, IL-1β and IL-8 expression in synovial fibroblasts and ameliorates cartilage degradation: implications for the treatment of rheumatoid arthritis. *Front Immunol, 12*, 790925. doi:10.3389/fimmu.2021.790925

Agbebi, E. A., Adeyemi, S. O., Adewale, A. I., Ajofoyinbo, O. S., Olugbogi, E. A., Oyinloye, O. M., . . . Oyinloye, B. E. (2025). Uvarinol and Dichamanetin derived from Uvaria chamae as potential dual-site inhibitors against PBP2a in methicillin resistant Staphylococcus aureus: An In silico study. *Pharmaceuticals (Basel), 18*(4). doi:10.3390/ph18040529

Anburajan, L., Meena, B., Vinithkumar, N. V., & Dharani, G. (2021). Molecular characterization of glycine betaine biosynthesis genes from deep sea halophilic bacteria, Bacillus atrophaeus NIOT-DSB21. *Ecological Genetics and Genomics, 18*, 100080. doi:<https://doi.org/10.1016/j.egg.2021.100080>

Angelini, L. L., Dos Santos, R. A. C., Fox, G., Paruthiyil, S., Gozzi, K., Shemesh, M., & Chai, Y. (2023). Pulcherrimin protects Bacillus subtilis against oxidative stress during biofilm development. *NPJ Biofilms Microbiomes, 9*(1), 50. doi:10.1038/s41522-023-00418-z

Barnes, V. M., Teles, R., Trivedi, H. M., Devizio, W., Xu, T., Mitchell, M. W., . . . Guo, L. (2009). Acceleration of purine degradation by periodontal diseases. *J Dent Res, 88*(9), 851-855. doi:10.1177/0022034509341967

Bryskier, A. (2005). Fusidic acid. *Antimicrobial Agents: Antibacterials and Antifungals*, 631-641.

Chaban, V. V., Nielsen, M. B., Kopec, W., & Khandelia, H. (2014). Insights into the role of cyclic ladderane lipids in bacteria from computer simulations. *Chem Phys Lipids, 181*, 76-82. doi:10.1016/j.chemphyslip.2014.04.002

Chang, Y., Chen, Y., Zhou, Q., Wang, C., Chen, L., Di, W., & Zhang, Y. (2020). Short-chain fatty acids accompanying changes in the gut microbiome contribute to the development of hypertension in patients with preeclampsia. *Clin Sci (Lond), 134*(2), 289-302. doi:10.1042/cs20191253

Chapple, I. L. C., Mealey, B. L., Van Dyke, T. E., Bartold, P. M., Dommisch, H., Eickholz, P., . . . Yoshie, H. (2018). Periodontal health and gingival diseases and conditions on an intact and a reduced periodontium: Consensus report of workgroup 1 of the 2017 World Workshop on the Classification of Periodontal and Peri-Implant Diseases and Conditions. *J Periodontol, 89 Suppl 1*, S74-s84. doi:10.1002/jper.17-0719

Chechetkin, I. R., Mukhitova, F. K., Blufard, A. S., Yarin, A. Y., Antsygina, L. L., & Grechkin, A. N. (2009). Unprecedented pathogen-inducible complex oxylipins from flax--linolipins A and B. *Febs j, 276*(16), 4463-4472. doi:10.1111/j.1742-4658.2009.07153.x

Chinthanom, P., Vichai, V., Rachtawee, P., Boonpratuang, T., & Isaka, M. (2023). Antimalarial lanostane dimers from artificially cultivated fruiting bodies of Ganoderma weberianum. *J Nat Prod, 86*(10), 2304-2314. doi:10.1021/acs.jnatprod.3c00457

Cho, K., Schwaiger-Haber, M., Naser, F. J., Stancliffe, E., Sindelar, M., & Patti, G. J. (2021). Targeting unique biological signals on the fly to improve MS/MS coverage and identification efficiency in metabolomics. *Anal Chim Acta, 1149*, 338210. doi:10.1016/j.aca.2021.338210

Chowdhury, S. K., Dutta, T., Chattopadhyay, A. P., Ghosh, N. N., Chowdhury, S., & Mandal, V. (2021). Isolation of antimicrobial Tridecanoic acid from Bacillus sp. LBF-01 and its potentialization through silver nanoparticles synthesis: a combined experimental and theoretical studies. *Journal of Nanostructure in Chemistry, 11*(4 (December 2021)). doi:10.1007/s40097-020-00385-3

Chrzanowski, Ł., Ławniczak, Ł., & Czaczyk, K. (2012). Why do microorganisms produce rhamnolipids? *World J Microbiol Biotechnol, 28*(2), 401-419. doi:10.1007/s11274-011-0854-8

Conroy, M. J., Andrews, R. M., Andrews, S., Cockayne, L., Dennis, E. A., Fahy, E., . . . O'Donnell, V. B. (2024). LIPID MAPS: update to databases and tools for the lipidomics community. *Nucleic Acids Res, 52*(D1), D1677-d1682. doi:10.1093/nar/gkad896

Danaher, M., Howells, L. C., Crooks, S. R., Cerkvenik-Flajs, V., & O'Keeffe, M. (2006). Review of methodology for the determination of macrocyclic lactone residues in biological matrices. *J Chromatogr B Analyt Technol Biomed Life Sci, 844*(2), 175-203. doi:10.1016/j.jchromb.2006.07.035

Daniel, J. M., Friess, S. D., Rajagopalan, S., Wendt, S., & Zenobi, R. (2002). Quantitative determination of noncovalent binding interactions using soft ionization mass spectrometry. *International Journal of Mass Spectrometry, 216*(1), 1-27. doi:<https://doi.org/10.1016/S1387-3806(02)00585-7>

Ding, J., Tan, L., Wu, L., Li, J., Zhang, Y., Shen, Z., . . . Gao, L. (2025). Regulation of tryptophan-indole metabolic pathway in Porphyromonas gingivalis virulence and microbiota dysbiosis in periodontitis. *NPJ Biofilms Microbiomes, 11*(1), 37. doi:10.1038/s41522-025-00669-y

Ding, L., Li, T., Liao, X., He, S., & Xu, S. (2018). Asperitaconic acids A-C, antibacterial itaconic acid derivatives produced by a marine-derived fungus of the genus Aspergillus. *J Antibiot (Tokyo), 71*(10), 902-904. doi:10.1038/s41429-018-0079-2

Eng, F., Marin, J. E., Zienkiewicz, K., Gutiérrez-Rojas, M., Favela-Torres, E., & Feussner, I. (2021). Jasmonic acid biosynthesis by fungi: derivatives, first evidence on biochemical pathways and culture conditions for production. *PeerJ, 9*, e10873. doi:10.7717/peerj.10873

Fazius, F., Shelest, E., Gebhardt, P., & Brock, M. (2012). The fungal α-aminoadipate pathway for lysine biosynthesis requires two enzymes of the aconitase family for the isomerization of homocitrate to homoisocitrate. *Mol Microbiol, 86*(6), 1508-1530. doi:10.1111/mmi.12076

Flentie, K. N., Stallings, C. L., Turk, J., Minnaard, A. J., & Hsu, F. F. (2016). Characterization of phthiocerol and phthiodiolone dimycocerosate esters of M. tuberculosis by multiple-stage linear ion-trap MS. *J Lipid Res, 57*(1), 142-155. doi:10.1194/jlr.D063735

Gu, P., Zhao, S., Li, C., Jiang, S., Zhou, H., & Li, Q. (2023). Construction of Recombinant Escherichia coli with a High L-Phenylalanine Production Yield from Glucose. *Microbiology Research, 14*(3), 1185-1198.

Hohmann, M., Brunner, V., Johannes, W., Schum, D., Carroll, L. M., Liu, T., . . . Gulder, T. A. M. (2024). Bacillamide D produced by Bacillus cereus from the mouse intestinal bacterial collection (miBC) is a potent cytotoxin in vitro. *Commun Biol, 7*(1), 655. doi:10.1038/s42003-024-06208-3

Hsieh, H. M., & Ju, Y. M. (2018). Medicinal components in Termitomyces mushrooms. *Appl Microbiol Biotechnol, 102*(12), 4987-4994. doi:10.1007/s00253-018-8991-8

Huang, C. B., Alimova, Y., Myers, T. M., & Ebersole, J. L. (2011). Short- and medium-chain fatty acids exhibit antimicrobial activity for oral microorganisms. *Arch Oral Biol, 56*(7), 650-654. doi:10.1016/j.archoralbio.2011.01.011

Hunter, W. N. (2007). The non-mevalonate pathway of isoprenoid precursor biosynthesis. *J Biol Chem, 282*(30), 21573-21577. doi:10.1074/jbc.R700005200

Ishihara, C., Sako, M., Tsutsumi, K., Fujii, N., Hashimoto, D., Sato, A., . . . Uchiyama, A. (2025). Involvement of propionate, citrulline, homoserine, and succinate in oral microbiome metabolite-driven periodontal disease progression. *Sci Rep, 15*(1), 7149. doi:10.1038/s41598-025-91105-w

Kawai, Y., Yano, I., & Kaneda, K. (1988). Various kinds of lipoamino acids including a novel serine-containing lipid in an opportunistic pathogen Flavobacterium. Their structures and biological activities on erythrocytes. *Eur J Biochem, 171*(1-2), 73-80. doi:10.1111/j.1432-1033.1988.tb13760.x

Kawaide, H. (2006). Biochemical and molecular analyses of gibberellin biosynthesis in fungi. *Biosci Biotechnol Biochem, 70*(3), 583-590. doi:10.1271/bbb.70.583

Kim, K. R., & Oh, D. K. (2013). Production of hydroxy fatty acids by microbial fatty acid-hydroxylation enzymes. *Biotechnol Adv, 31*(8), 1473-1485. doi:10.1016/j.biotechadv.2013.07.004

Kobayashi, S., Tamura, T., Koshishiba, M., Yasumoto, T., Shimizu, S., Kintaka, T., & Nagai, K. (2021). Total synthesis, structure revision, and neuroprotective effect of hericenones C-H and their derivatives. *J Org Chem, 86*(3), 2602-2620. doi:10.1021/acs.joc.0c02681

Koilybayeva, M., Shynykul, Z., Ustenova, G., Waleron, K., Jońca, J., Mustafina, K., . . . Alibayeva, Z. (2023). Gas chromatography-mass spectrometry profiling of volatile metabolites produced by some Bacillus spp. and evaluation of their antibacterial and antibiotic activities. *Molecules, 28*(22). doi:10.3390/molecules28227556

Lee, J. H., Kim, Y. G., Khadke, S. K., & Lee, J. (2021). Antibiofilm and antifungal activities of medium-chain fatty acids against Candida albicans via mimicking of the quorum-sensing molecule farnesol. *Microb Biotechnol, 14*(4), 1353-1366. doi:10.1111/1751-7915.13710

Li, X., Zhang, B., Hu, Y., & Zhao, Y. (2021). New insights into gut-bacteria-derived indole and its derivatives in intestinal and liver diseases. *Front Pharmacol, 12*, 769501. doi:10.3389/fphar.2021.769501

Ma, F., Wang, J., Jiang, W., Luo, J., Yang, R., Zhang, L., & Han, C. (2024). Ganoderic acid A: a potential natural neuroprotective agent for neurological disorders: a review. *Int J Med Mushrooms, 26*(2), 11-23. doi:10.1615/IntJMedMushrooms.2023051918

Mansour, M. A. K., Ali, S. G., Hassan, M. A. M., Gabra, F. A., & Mawad, A. M. M. (2025). Optimization of citrulline production from a Bacillus subtilis BH-01 isolated from raw buffalo milk. *BMC Microbiol, 25*(1), 71. doi:10.1186/s12866-025-03768-0

Matsumaru, T., Sunazuka, T., Hirose, T., Ishiyama, A., Namatame, M., Fukuda, T., . . . Ōmura, S. (2008). Synthesis and biological properties of tensyuic acids B, C, and E, and investigation of the optical purity of natural tensyuic acid B. *Tetrahedron, 64*(30), 7369-7377. doi:<https://doi.org/10.1016/j.tet.2008.05.035>

Meguro, A., Tomita, T., Nishiyama, M., & Kuzuyama, T. (2013). Identification and characterization of bacterial diterpene cyclases that synthesize the cembrane skeleton. *Chembiochem, 14*(3), 316-321. doi:10.1002/cbic.201200651

Miersch, O., Schneider, G., & Sembdner, G. (1991). Hydroxylated jasmonic acid and related compounds from Botryodiplodia theobromae. *Phytochemistry, 30*(12), 4049-4051. doi:<https://doi.org/10.1016/0031-9422(91)83464-V>

Moon, J. H., Herr, Y., Kim, S. W., & Lee, J. Y. (2011). In vitro activity of deferoxamine against Porphyromonas gingivalis. *FEMS Microbiol Lett, 323*(1), 61-67. doi:10.1111/j.1574-6968.2011.02357.x

Nara, F., Tanaka, M., Masuda-Inoue, S., Yamasato, Y., Doi-Yoshioka, H., Suzuki-Konagai, K., . . . Ogita, T. (1999). Biological activities of scyphostatin, a neutral sphingomyelinase inhibitor from a discomycete, Trichopeziza mollissima. *J Antibiot (Tokyo), 52*(6), 531-535. doi:10.7164/antibiotics.52.531

Nemoto, T., Ojika, M., Takahata, Y., Andoh, T., & Sakagami, Y. (1998). Structures of topostins, DNA topoisomerase I inhibitors of bacterial origin. *Tetrahedron, 54*(12), 2683-2690. doi:<https://doi.org/10.1016/S0040-4020(98)83004-4>

Ngoc, N. T., Hanh, T. T. H., Quang, T. H., Cuong, N. X., Nam, N. H., Thao, D. T., . . . Minh, C. V. (2021). Polyhydroxylated steroids from the Vietnamese soft coral Sarcophyton ehrenbergi. *Steroids, 176*, 108932. doi:10.1016/j.steroids.2021.108932

Noguchi, M., Shimizu, M., Lu, P., Takahashi, Y., Yamauchi, Y., Sato, S., . . . Sato, R. (2022). Lactic acid bacteria-derived γ-linolenic acid metabolites are PPARδ ligands that reduce lipid accumulation in human intestinal organoids. *J Biol Chem, 298*(11), 102534. doi:10.1016/j.jbc.2022.102534

Omura, S. (1981). Cerulenin. *Methods Enzymol, 72*, 520-532.

Oshkin, I. Y., Tikhonova, E. N., Suleimanov, R. Z., Ashikhmin, A. A., Ivanova, A. A., Pimenov, N. V., & Dedysh, S. N. (2023). All kinds of sunny colors synthesized from methane: genome-encoded carotenoid production by methylomonas species. *Microorganisms, 11*(12). doi:10.3390/microorganisms11122865

Parsons, C. V., Harris, D. M., & Patten, C. L. (2015). Regulation of indole-3-acetic acid biosynthesis by branched-chain amino acids in Enterobacter cloacae UW5. *FEMS Microbiol Lett, 362*(18), fnv153. doi:10.1093/femsle/fnv153

Phimister, A. J., Lee, M. G., Morin, D., Buckpitt, A. R., & Plopper, C. G. (2004). Glutathione depletion is a major determinant of inhaled naphthalene respiratory toxicity and naphthalene metabolism in mice. *Toxicol Sci, 82*(1), 268-278. doi:10.1093/toxsci/kfh258

Raetz, C. R., & Whitfield, C. (2002). Lipopolysaccharide endotoxins. *Annu Rev Biochem, 71*, 635-700. doi:10.1146/annurev.biochem.71.110601.135414

Reijneveld, J. F., Marino, L., Cao, T. P., Cheng, T. Y., Dam, D., Shahine, A., . . . Van Rhijn, I. (2021). Rational design of a hydrolysis-resistant mycobacterial phosphoglycolipid antigen presented by CD1c to T cells. *J Biol Chem, 297*(4), 101197. doi:10.1016/j.jbc.2021.101197

Roser, L. A., Erkoc, P., Ingelfinger, R., Henke, M., Ulshöfer, T., Schneider, A. K., . . . Schiffmann, S. (2022). Lecanoric acid mediates anti-proliferative effects by an M phase arrest in colon cancer cells. *Biomed Pharmacother, 148*, 112734. doi:10.1016/j.biopha.2022.112734

Ryan, E., Joyce, S. A., & Clarke, D. J. (2023). Membrane lipids from gut microbiome-associated bacteria as structural and signalling molecules. *Microbiology (Reading), 169*(3). doi:10.1099/mic.0.001315

Sekowska, A., Ashida, H., & Danchin, A. (2019). Revisiting the methionine salvage pathway and its paralogues. *Microb Biotechnol, 12*(1), 77-97. doi:10.1111/1751-7915.13324

Sepe, V., Ummarino, R., D'Auria, M. V., Chini, M. G., Bifulco, G., Renga, B., . . . Zampella, A. (2012). Conicasterol E, a small heterodimer partner sparing farnesoid X receptor modulator endowed with a pregnane X receptor agonistic activity, from the marine sponge Theonella swinhoei. *J Med Chem, 55*(1), 84-93. doi:10.1021/jm201004p

Shi, Y., Ji, M., Dong, J., Shi, D., Wang, Y., Liu, L., . . . Liu, L. (2024). New bioactive secondary metabolites from fungi: 2023. *Mycology, 15*(3), 283-321. doi:10.1080/21501203.2024.2354302

Shrestha, P., Karmacharya, J., Han, S. R., Lee, J. H., & Oh, T. J. (2024). Elucidation of bacterial trehalose-degrading trehalase and trehalose phosphorylase: physiological significance and its potential applications. *Glycobiology, 34*(2). doi:10.1093/glycob/cwad084

Stancliffe, E., & Patti, G. J. (2023). PeakDetective: A Semisupervised Deep Learning-Based Approach for Peak Curation in Untargeted Metabolomics. *Anal Chem, 95*(25), 9397-9403. doi:10.1021/acs.analchem.3c00764

Stancliffe, E., Schwaiger-Haber, M., Sindelar, M., Murphy, M. J., Soerensen, M., & Patti, G. J. (2022). An Untargeted Metabolomics Workflow that Scales to Thousands of Samples for Population-Based Studies. *Anal Chem, 94*(50), 17370-17378. doi:10.1021/acs.analchem.2c01270

Stancliffe, E., Schwaiger-Haber, M., Sindelar, M., & Patti, G. J. (2021). DecoID improves identification rates in metabolomics through database-assisted MS/MS deconvolution. *Nat Methods, 18*(7), 779-787. doi:10.1038/s41592-021-01195-3

Taba, M., Jr., Kinney, J., Kim, A. S., & Giannobile, W. V. (2005). Diagnostic biomarkers for oral and periodontal diseases. *Dent Clin North Am, 49*(3), 551-571, vi. doi:10.1016/j.cden.2005.03.009

Takaichi, S., & Shimada, K. (1999). Pigment composition of two pigment-protein complexes derived from anaerobically and semi-aerobically grown Rubrivivax gelatinosus, and identification of a new keto-carotenoid, 2-ketospirilloxanthin1. *Plant and Cell Physiology, 40*(6), 613-617. doi:10.1093/oxfordjournals.pcp.a029584

Tempesta, M. S., Kriek, G. R., & Bates, R. B. (1982). Uvaricin, a new antitumor agent from Uvaria accuminata (Annonaceae). *The Journal of Organic Chemistry, 47*(16), 3151-3153. doi:10.1021/jo00137a024

Thai, V. C., Stubbs, K. A., Sarkar-Tyson, M., & Kahler, C. M. (2023). Phosphoethanolamine transferases as drug discovery targets for therapeutic treatment of multi-drug resistant pathogenic gram-negative bacteria. *Antibiotics (Basel), 12*(9). doi:10.3390/antibiotics12091382

Tørring, T., Shames, S. R., Cho, W., Roy, C. R., & Crawford, J. M. (2017). Acyl histidines: new N-acyl amides from Legionella pneumophila. *Chembiochem, 18*(7), 638-646. doi:10.1002/cbic.201600618

Wishart, D. S., Li, C., Marcu, A., Badran, H., Pon, A., Budinski, Z., . . . Ramirez-Gaona, M. (2020). PathBank: a comprehensive pathway database for model organisms. *Nucleic Acids Res, 48*(D1), D470-d478. doi:10.1093/nar/gkz861

Xu, H., Andi, B., Qian, J., West, A. H., & Cook, P. F. (2006). The alpha-aminoadipate pathway for lysine biosynthesis in fungi. *Cell Biochem Biophys, 46*(1), 43-64. doi:10.1385/cbb:46:1:43

Zhao, Z. Z., Guo, L., Shan, W., Chu, C. H., & Zhang, J. (2024). Silent signals: how N-acyl homoserine lactones drive oral microbial behaviour and health outcomes. *Front Oral Health, 5*, 1484005. doi:10.3389/froh.2024.1484005

Zheng, H., Tu, Y., Ning, X., Guo, Q., Ren, B., Xie, J., & Liu, C. (2025). The microbial metabolite isovaleric acid aggravates gelatinase-mediated periodontal tissue destruction via the NF-κB signaling pathway. *J Periodontol*. doi:10.1002/jper.11375

Zhou, T. C., Kim, B. G., & Zhong, J. J. (2014). Enhanced production of validamycin A in Streptomyces hygroscopicus 5008 by engineering validamycin biosynthetic gene cluster. *Appl Microbiol Biotechnol, 98*(18), 7911-7922. doi:10.1007/s00253-014-5943-9
